# Promoting Hand Hygiene During the COVID-19 Pandemic: Parallel Randomized Trial for the Optimization of the Soapp App

**DOI:** 10.2196/43241

**Published:** 2023-02-03

**Authors:** Dario Baretta, Melanie Alexandra Amrein, Carole Bäder, Gian Giacomo Ruschetti, Carole Rüttimann, Maria Del Rio Carral, Carlo Fabian, Jennifer Inauen

**Affiliations:** 1 Institute of Psychology University of Bern Bern Switzerland; 2 Institute of Psychology University of Lausanne Lausanne Switzerland; 3 Institute for Social Work and Health FHNW School of Social Work Olten Switzerland

**Keywords:** COVID-19, hand hygiene, behavior change intervention, Multiphase Optimization Strategy, MOST, smartphone apps, motivation, habit, social norm, mobile phone

## Abstract

**Background:**

Hand hygiene is an effective behavior for preventing the spread of the respiratory disease COVID-19 and was included in public health guidelines worldwide. Behavior change interventions addressing hand hygiene have the potential to support the adherence to public health recommendations and, thereby, prevent the spread of COVID-19. However, randomized trials are largely absent during a pandemic; therefore, there is little knowledge about the most effective strategies to promote hand hygiene during an ongoing pandemic. This study addresses this gap by presenting the results of the optimization phase of a Multiphase Optimization Strategy of *Soapp*, a smartphone app for promoting hand hygiene in the context of the COVID-19 pandemic.

**Objective:**

This study aimed to identify the most effective combination and sequence of 3 theory- and evidence-based intervention modules (habit, motivation, and social norms) for promoting hand hygiene. To this end, 9 versions of *Soapp* were developed (conditions), and 2 optimization criteria were defined: the condition with the largest increase in hand hygiene at follow-up and condition with the highest engagement, usability, and satisfaction based on quantitative and qualitative analyses.

**Methods:**

This study was a parallel randomized trial with 9 intervention conditions defined by the combination of 2 intervention modules and their sequence. The trial was conducted from March to August 2021 with interested participants from the Swiss general population (N=232; randomized). Randomization was performed using Qualtrics (Qualtrics International Inc), and blinding was ensured. The duration of the intervention was 34 days. The primary outcome was self-reported hand hygiene at follow-up, which was assessed using an electronic diary. The secondary outcomes were user engagement, usability, and satisfaction assessed at follow-up. Nine participants were further invited to participate in semistructured exit interviews. A set of ANOVAs was performed to test the main hypotheses, whereas a thematic analysis was performed to analyze the qualitative data.

**Results:**

The results showed a significant increase in hand hygiene over time across all conditions. There was no interaction effect between time and intervention condition. Similarly, no between-group differences in engagement, usability, and satisfaction emerged. Seven themes (eg, “variety and timeliness of the task load” and “social interaction”) were found in the thematic analysis.

**Conclusions:**

The effectiveness of *Soapp* in promoting hand hygiene laid the foundation for the next evaluation phase of the app. More generally, the study supported the value of digital interventions in pandemic contexts. The findings showed no differential effect of intervention conditions involving different combinations and sequences of the habit, motivation, and social norms modules on hand hygiene, engagement, usability, and satisfaction. In the absence of quantitative differences, we relied on the results from the thematic analysis to select the best version of *Soapp* for the evaluation phase.

**Trial Registration:**

ClinicalTrials.gov NCT04830761; https://clinicaltrials.gov/ct2/show/NCT04830761

**International Registered Report Identifier (IRRID):**

RR2-10.1136/bmjopen-2021-055971

## Introduction

### Background

Hand hygiene is an effective behavior for decreasing the transmission of respiratory illnesses [1,2], including COVID-19 [3]. Therefore, recommendations to perform correct hand hygiene at key times have been included in public health guidelines worldwide to counter the spread of COVID-19 [4]. To facilitate the adoption of public health guidelines, the development and evaluation of effective behavior change interventions was identified as a priority of the COVID-19 research agenda, in particular, owing to the fact that limited or no contextualized evidence was available on the effectiveness of behavior change interventions during pandemics [5]. Although evidence synthesis reports became available during the COVID-19 pandemic (July to December 2020), showing a medium, positive effect of hand hygiene interventions developed to counter the spread of various respiratory viruses (eg, influenza virus, respiratory syncytial virus, and adenovirus) [6], their validity and relevance to the COVID-19 pandemic can be questioned. For example, the reviewed studies included interventions targeted at diverse respiratory infections that did not cause pandemics (eg, influenza, flu, and cold) or lead to the spread of a pandemic of the same magnitude as that of the COVID-19 pandemic (eg, pandemic influenza A H1N1).

The need for research on effective behavior change interventions for promoting hand hygiene during a pandemic was further confirmed by the fluctuation in hand hygiene over the course of the COVID-19 pandemic. At first, results indicated high adherence among the public. During the first wave of the pandemic (ie, between March and May 2020), studies suggested that (1) hand hygiene was one of the most adopted protective behaviors against the spread of COVID-19 [7], and (2) the frequency and correctness of hand hygiene behavior in key situations (ie, after coughing, sneezing, blowing one’s nose and upon reaching home or workplace) improved compared with the period before the pandemic outbreak [8,9]. However, research including longer periods of the pandemic showed a decrease in hand hygiene over time. For example, a study conducted from May 2020 to August 2021 showed that almost one-third of the adults from the general population did not comply with hand hygiene recommendations, and some of them had no intention to change their behavior [10]. In addition, there is evidence of significant associations between hand hygiene and indicators of the pandemic trajectory (eg, the increase in recent cases of COVID-19 morbidity is associated with an increase in the frequency of self-reported hand hygiene) [11]. Taken together, the literature suggests that hand hygiene is not consistently performed throughout a pandemic and is prone to variations over time. Therefore, fostering sustained hand hygiene through effective behavior change interventions represented a public health priority to counter the spread of COVID-19 and future pandemics.

During an ongoing pandemic in which social contact should be limited, digital interventions have the advantage that no personal contact is required for their use; moreover, they can be personalized and potentially be integrated into the daily lives of an unlimited number of people. Interventions based on smartphone apps can deliver behavior change techniques [12] in real life that could lead to substantial population-level impact and long-term health behavior change [13]. However, recent reviews have pointed out that there is limited knowledge about how to effectively promote hand hygiene using digital interventions in the general population [6,14].

To address these research gaps, we devised a Multiphase Optimization Strategy (MOST) [15] to develop and test Soapp, an effective smartphone-based behavior change intervention for promoting hand hygiene during the ongoing COVID-19 pandemic [16]. In the preparation phase, we developed 3 intervention modules—tackling habit, motivation, and social norms—based on behavior change theory and empirical evidence [16].

### Aim of This Study

This study focused on the optimization phase of Soapp, which aimed to identify the most effective combination and sequence of the developed intervention modules to be included in the subsequent evaluation phase. As described in the study protocol [16], during the optimization phase, we compared 9 different combinations of the 3 developed modules (ie, habit, motivation, and social norms). Overall, 2 optimization criteria were defined to select the best intervention version for the subsequent evaluation phase. The optimization criteria were as follows: (1) the condition with the largest increase in hand hygiene at key times at follow-up (T3) and (2) the condition with the highest engagement, usability, and satisfaction. Regarding the first criterion, we tested the following preregistered hypotheses [16]:

Hypothesis (H) 1; H1: The intervention groups show a significant increase in correct hand hygiene at key times after 4 weeks (T3) of intervention compared with baseline (T1).

H2: The intervention groups significantly differ in the effects of the intervention on correct hand hygiene behavior at key times (T1-T3).

In case of significant between-group differences in hand hygiene at key times, post hoc tests were performed to determine the most effective condition. In addition, we investigated the unique contribution of each module by testing the following hypotheses that were not preregistered:

H3: The intervention groups with the *habit* module show a significant increase in correct hand hygiene behavior at key times (T1-T3) compared with the groups without the *habit* module.

H4: The intervention groups with the *motivation* module show a significant increase in correct hand hygiene behavior at key times (T1-T3) compared with the groups without the *motivation* module.

H5: The intervention groups with the *social* module show a significant increase in correct hand hygiene behavior at key times (T1-T3) compared with the groups without the *social* module.

The second optimization criterion leveraged a combination of quantitative and qualitative methods to explore the participants’ engagement and satisfaction with the app as well as its usability. This criterion was tested using the following hypotheses:

The intervention groups significantly differ in the engagement with (H6), usability of (H7), and satisfaction (H8) with the intervention (T3).

In addition, semistructured interviews were conducted to explore which aspects and features of Soapp were perceived as more usable and more important for supporting engagement and satisfactory experiences after 34 days of using the app.

As secondary outcomes, we had preregistered a series of hypotheses regarding the psychological mechanisms and health impact of the intervention that did not inform the optimization decision. We have reported these in Multimedia Appendix 1 for completion.

## Methods

### Study Design

**Figure 1:**
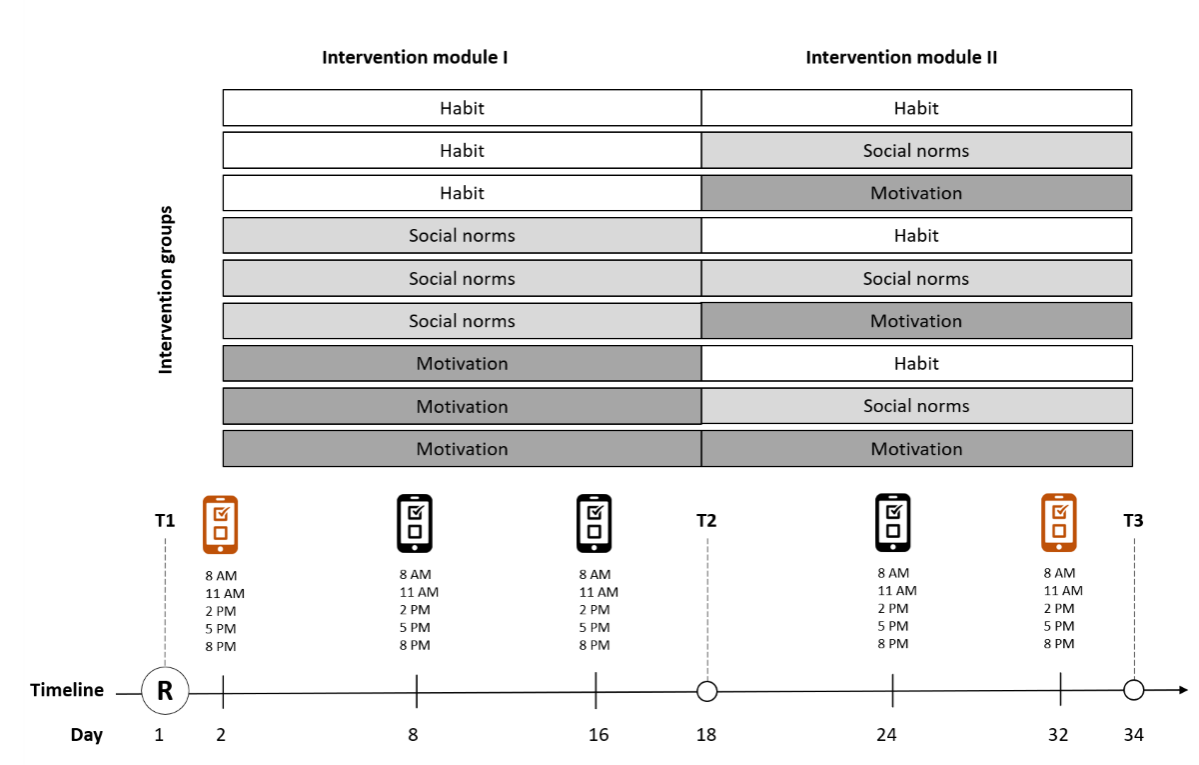
Intervention optimization. Red diaries represent baseline (T1) and follow-up (T3) assessments for the primary outcome (hand hygiene). R: randomization.

The study design for the optimization phase was a double-blind parallel randomized trial. The participants were randomized to 1 of 9 intervention groups in a 1:1:1:1:1:1:1:1:1 ratio and completed 2 consecutive intervention modules, as shown in Figure 1. The total duration of the optimization study (ie, recruitment and data collection) was set to 6 months (start: March 26, 2021) or until a total of 465 participants were enrolled, whichever came first. The duration of the optimization trial for each participant (ie, time between T1 and T3) was 34 days. At the end of the study, as a part of the second optimization criterion, qualitative interviews were conducted with a subsample to collect in-depth information about the engagement with, usability of, and satisfaction with the intervention.

### Ethics Approval

This trial is registered at ClinicalTrials.gov (NCT04830761), and the reporting is in line with the CONSORT (Consolidated Standards of Reporting Trials) guidelines [17] (Multimedia Appendix 2). The trial received ethics approval from the Swiss Ethics Committee of the Canton of Bern (ID: 2021-00164).

### Participants

The target population for the Soapp app was German-speaking adults from the Swiss population who were interested in using an app to improve hand hygiene behavior. The inclusion criteria were as follows: (1) being aged at least 18 years, (2) owning a smartphone with mobile access to the internet, (3) being proficient in the German language, and (4) having signed an electronic informed consent form to participate in the study. As presented in the study protocol [16], the initial target sample size for the optimization phase was 387 participants. The sample size was calculated to perform a repeated-measures ANOVA with a within (time: T1-T3)-between (intervention group) interaction. The sample size was determined using an a priori power analysis with G*Power (Heinrich-Heine-Universität Düsseldorf) [18] (β=.80; α=.05; *F*_8_=0.1). Assuming a 20% attrition during the course of the intervention, the target sample size for the study was raised to 465 participants. However, owing to both trial and project timelines, we stopped recruiting after 5 months for a total study duration of 6 months.

A subsample (n=9) participated in qualitative interviews. The recruitment was based on the participants’ willingness to participate in semistructured interviews, as assessed at the end of the last survey (T3). The aim of the qualitative interview was to recruit an even number of participants per intervention module according to hand hygiene adherence: low adherence, medium adherence, and high adherence to hand hygiene. The participants in the ≤33rd percentile were assigned to the low adherence group (3/9, 33%), participants in between the 34th and 66th percentiles were assigned to the medium adherence group (3/9, 33%), and participants in the ≥67th percentile were assigned to the high adherence group (3/9, 33%). The sample size (n=9) was smaller than that reported in the study protocol (n=15) because the recruitment was stopped when theoretical saturation was achieved (ie, no new themes emerged) [19].

### Outcomes

#### Primary Outcome

The primary outcome of the study (ie, the first optimization criterion), the frequency of correct hand hygiene at key times at T3, was assessed via ecological momentary assessment with the support of an electronic diary embedded in Soapp. On diary days (days 2, 8, 16, 24, and 32), the participants were prompted 5 times per day to indicate whether each of the 13 key times to perform hand hygiene defined by the Swiss Federal Office of Public Health had occurred (eg, upon arriving home and after using the toilet; Multimedia Appendix 1). For each situation that occurred, the participants were asked how often they correctly washed or disinfected their hands in that specific situation. The 5-point response scale ranged from never (1) to always (5). The main outcome was operationalized as the mean reported frequency of correct hand hygiene across all the indicated key times and ranged from 1 to 5. To test the H1 and H2, the assessment points considered for hand hygiene behavior were the first diary filled out on day 2 (T1) and the last diary filled out on day 32 (T3).

#### Secondary Outcomes

Engagement, usability, and satisfaction (ie, the second optimization criterion) were measured at T3. User engagement was assessed using the digital behavior change interventions (DBCI) Engagement Scale [20], a 7-point Likert scale ranging from *not at all* (1) to *extremely* (7; Cronbach α=.78). Intervention usability was assessed using the System Usability Scale [21], a 6-point Likert scale ranging from *I do not agree at all* (1) to *I agree completely* (6; Cronbach α=.80). Satisfaction was assessed using the Fragebogen zur Messung der Patientenzufriedenheit (ZUF)-8 [22], a 4-point Likert scale ranging from 0 to 3 (Cronbach α=.89).

Other variables assessed during the study but not relevant to the current report are described in the clinical trial registration, and the corresponding results are presented in Multimedia Appendix 1.

### Procedure

The participants were recruited via social media (eg, Facebook [Meta Platforms, Inc] and Instagram [Meta Platforms, Inc]), mailing lists, and leaflets with the help of a market research company and with the aim of recruiting a diverse range of people from the German-speaking adult Swiss general population in terms of gender, age, and socioeconomic status. Interested people who clicked on the campaign link were led to a landing page with the study information. Those who chose to continue were redirected to the study page on REDCap (Research Electronic Data Capture; Vanderbilt University) [23] where they could read and watch a video of the study information, fill out an eligibility and consent survey, and sign the e-consent form. After providing electronic informed consent to participate in the study, the participants received a registration code via email and were guided to download the Soapp app from iTunes (Apple Inc) or Google Play Store (Google LLC) and register on it. The day after the registration, the participants received the T1 questionnaire and were then randomized into one of the intervention groups. Randomization was implemented in Qualtrics (Qualtrics International Inc), which preserved the allocation concealment. In addition, the researchers involved in the study were blinded to the intervention assignment, as the participant identifier was pseudoanonymized before randomization. The day after the randomization, the participants filled out the first-hand hygiene diary. The diary included five 1-minute questionnaires per day to avoid retrospective bias in reporting hand hygiene [24].

The optimization trial lasted 34 days and included two 2-week intervention modules (Figure 1). During the first module, the participants filled out the hand hygiene diary on days 2, 8, and 16. After the first module, the participants received a second questionnaire (T2) and a second intervention module, which followed the same structure as the first. After completing the T2, the participants were offered a small gift (ie, a bar of hand soap and a thank you card) to prevent attrition, which was sent to their homes. During the second intervention module, they filled out the hand hygiene diaries on days 24 and 32. At the end of the second module, the participants received the final questionnaire (T3). The participants were given the chance to win 1 of 3 iPhones (Apple Inc) 12s after both the optimization and evaluation phases of the study were completed. The questionnaires and diaries were integrated into Qualtrics services using Soapp’s application programming interface, and the participants’ data were stored on Qualtrics.

The participants who were given the option and volunteered to participate in the qualitative study were interviewed via telephone by a study team member (CB). This 30-minute interview was recorded and included questions about the usability of the app and the overall experience with the intervention modules in terms of satisfaction and engagement (Multimedia Appendix 3).

### Intervention

In the optimization phase, each arm of the parallel randomized trial was characterized by a unique combination and sequence of 2 of the 3 intervention modules: motivation, habit, and social norms (Figure 1). The modules were defined as the outcome of the preparation phase in which a theory- and evidence-based approach was followed. The resulting content is synthesized in Table 1 and detailed in supplemental material 1 of the protocol paper [16].

**Table 1:** Contents of the modules.

Module, TDF^a^ domain, and behavioral predictor			Behavior change technique
**Basic**			
	**Goals**		
		Intention	1.1 Goal setting (behavior)
	**Skills**		
		Skills	4.1 Instruction on how to perform behavior
	**Knowledge**		
		Knowledge	5.1 Information about health consequences
	**Environmental context and resources**		
		Resources and material resources (availability and management)	1.4 Action planning
**Motivation**			
	**Goals**		
		Intention	1.1 Goal setting (behavior)
	**Beliefs about consequences**		
		Risk perception	5.1 Information about health consequences
		Attitude	5.2 Salience of consequences
		Outcome expectancies	9.2 Pros and cons
		Intention	5.2 Salience of consequences
	**Beliefs about capabilities**		
		Self-efficacy	1.2 Problem solving 15.1 Verbal persuasion about capabilities 15.3 Focus on past success
	**Reinforcement**		
		Intention	10.9 Self-reward
**Habit**			
	**Knowledge**		
		Knowledge	4.2 Information about antecedents
	**Memory, attention, and decision processes**		
		Action control	2.3 Self-monitoring of behavior
	**Goals**		
		Action planning	1.4 Action planning 7.1 Prompts and cues
	**Skills and goals**		
		Habit strength	8.1 Behavioral practice and rehearsal 8.3 Habit formation
	**Behavioral regulation**		
		Habit strength	7.1 Prompts and cues (physical cue)
**Social norms**			
	**Social influences**		
		Descriptive norm	2.1 Monitoring of behavior by others without feedback 2.2 Feedback on behavior 6.2 Social comparison 10.4 Social reward 10.5 Social incentive
		Injunctive norm	5.1 Information about health consequences 6.3 Information about others’ approval 9.1 Credible source 10.5 Social incentive 12.1 Restructuring the physical environment

^a^TDF: Theoretical Domain Framework.

The modules were delivered to the participants via their personal smartphones through the study app Soapp. They were comparable in terms of user time and extent of content, and each module took 2 weeks to be completed. In addition, each intervention condition included a basic module that provided information on hand hygiene to all the participants. During the configuration process, the Soapp app underwent various iterative testing cycles to refine the content of each module and improve usability. The Soapp app contained all the information required to use it, and there was no direct contact with the study team.

### Data Analysis

#### Handling of Missing Data

Missing data were handled according to the intention-to-treat (ITT) principle [25]. The ITT analysis included all randomized participants. It ignores noncompliance, protocol deviations from the intervention modules, and anything that occurs after randomization. ITT analysis avoids overoptimistic estimates of the efficacy of an intervention resulting from the removal of noncompliers by accepting that noncompliance and protocol deviations that are likely to occur in practice. In line with previous research [26], missing data for hand hygiene behavior were replaced using the last observation carried forward approach.

#### Hypothesis Testing

To test the hypotheses related to the first optimization criterion, a repeated-measures ANOVA with a within-between interaction was used. The *within* effect was represented by the difference in hand hygiene between T1 and T3 (H1), whereas the *within-between* interaction was represented by the change in correct hand hygiene behavior between T1 and T3 across all 9 intervention groups (H2). If the groups differed significantly, post hoc tests were performed to identify the most effective intervention group. To test hypotheses H3, H4, and H5, 3 dummy variables were created: *habit exposure*, *motivation exposure*, and *social exposure.* These variables indicated whether a participant was exposed to the corresponding module during the intervention. Then, 3 repeated-measures ANOVAs, 1 for each dummy variable, with a within-between interaction were performed. Each ANOVA tested the interaction between time and the exposure to a specific module (Table 2). Finally, for the second optimization criterion, three 1-way ANOVAs were performed to test differences across conditions at T3 in terms of engagement (H6), usability (H7), and satisfaction (H8). For all the hypotheses, a set of sensitivity analyses with robust and nonparametric (ie, Kruskal-Wallis test) ANOVAs was performed to account for potential unequal sample sizes and nonnormal distribution of the data.

**Table 2:** Summary of hypothesis tests.

H^a^		Preregistered	Dependent variable	Within factor (time)	Between factor
**First optimization criterion based on the primary outcome**					
	H1	Yes	Hand hygiene	T1-T3	N/A^b^
	H2	Yes	Hand hygiene	T1-T3	Intervention groups
	H3	No	Hand hygiene	T1-T3	Habit exposure
	H4	No	Hand hygiene	T1-T3	Motivation exposure
	H5	No	Hand hygiene	T1-T3	Social exposure
**Second optimization criterion based on the secondary outcomes**					
	H6	No	Engagement	N/A	Intervention groups
	H7	No	Usability	N/A	Intervention groups
	H8	No	Satisfaction	N/A	Intervention groups

^a^H: hypothesis.

^b^N/A: not applicable.

#### Analytical Software

The packages *ez* and *WRS2* from the statistical software R (version 4.1.2; R Foundation for Statistical Computing) were used to perform parametric and robust ANOVAs, respectively. The data and R code used for the main analyses are available on the Open Science Framework repository platform [27].

#### Qualitative Analysis

Postintervention user engagement, usability, and satisfaction were explored using semistructured interviews. The interviews were transcribed verbatim, and the transcripts were analyzed using thematic analysis [28]. Thematic analysis is characterized by six phases: (1) familiarizing oneself with the data, (2) generating initial codes, (3) searching for themes, (4) reviewing themes, (5) defining and naming themes, and (6) producing the report. Data and repeated patterns that were considered pertinent to the aims of the study were coded by a coauthor (CR). New inductive codes were labeled as they were identified during the coding process, and the results of the coding process were iteratively discussed by 2 coauthors (CR and JI). The next stage involved searching for themes; CR reviewed the codes one by one, organizing the findings to combine different codes that focus on similar aspects. The ordered data were reviewed and revised in discussion among 3 coauthors (CR, JI, and DB) and were subsequently organized into themes. The resolution of disagreements and agreement on the final themes was achieved through discussion among CR, JI, and DB. After defining and naming the themes, examples of relevant transcripts were selected to illustrate them. The data were analyzed in their original language to preserve their original meanings. Illustrative quotes were translated by CR. Conclusions were drawn on the possible improvement of Soapp to optimize the effectiveness and usability of the intervention for the evaluation phase.

## Results

### Overview

**Figure 2:**
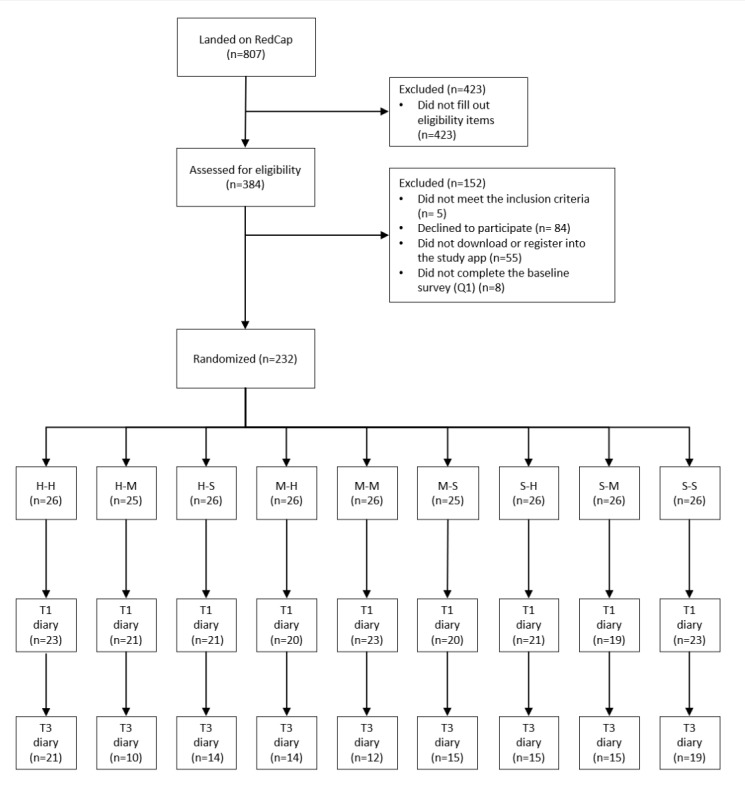
Participant recruitment flow. The intervention groups are specified as follows: H-H: habit-habit; H-M: habit-motivation; H-S: habit-social; M-H: motivation-habit; M-M: motivation-motivation; M-S: motivation-social; S-H: social-habit; S-M: social-motivation; and S-S: social-social. T1: baseline: T3: follow-up.

The recruitment for the optimization trial began on March 27, 2021, and ended on July 28, 2021. T3 data were collected between April 29 and August 25, 2021. Because of both trial and project timelines, we stopped the trial 6 months after the start of the study, with the recruitment lasting for 5 months. Overall, 232 participants were recruited and randomized into 1 of the 9 intervention conditions. Among these 232 participants, 14 (6%) participants did not fill out any of the 5 hand hygiene diaries, whereas 27 (11.6%) participants did not complete the first diary at T1. Another (1/232, 0.4%) participant completed the first diary but did not encounter any of the key situations to perform hand hygiene during that day. Therefore, these (42/232, 18.1%) participants were excluded from the analysis because the main outcome (ie, hand hygiene) at T1 was missing. Of the 232 participants who were randomized, 190 (81.9%) filled out the hand hygiene diary at T1, and 118 (62.1%; 50.9% of the randomized participants) of them filled out the hand hygiene diary at T3. Figure 2 shows the participants’ flow through randomization, T1 diary assessment, and T3 diary assessment for each intervention group. For the secondary analysis, we included only those participants who filled out the T3 panel assessment because the dependent variables were assessed only at T3.

### T1 Characteristics

Sociodemographic and hand hygiene behaviors at T1 are reported in Multimedia Appendix 4. The figures refer to the 190 participants who filled out the first diary at T1. The mean age of the participants was 39.9 (SD 15.9) years. Of the 190 participants, 139 (73.2%) were women, 125 (65.8%) had high school qualifications, 101 (53.2%) were employed, and 49 (25.8%) were living alone. Descriptive statistics for hand hygiene behavior (mean 4.01, SD 0.82; median=4.17; skewness=−1.24) suggested that hand hygiene behavior was already high at T1, with a moderate left-tailed distribution.

Dropout analysis was performed to investigate T1 differences between the participants who completed the study and those who dropped out at any point during the intervention. We analyzed all the 232 randomized participants, and those who did not complete the last panel assessment at T3 were categorized as dropouts (n=83, 36%). The results suggested no T1 differences between dropouts and retainers with respect to age (*F*_1,230_=2.17; *P*=.14), sex (*χ*^2^_1_=0.4; *P*=.55), hand hygiene (*F*_1,229_=0.24; *P*=.63), or intention to increase hand hygiene behavior (*F*_1,230_=0.72; *P*=.40).

### First Optimization Criterion: Change in Hand Hygiene Behavior

The main effects of time and the interaction between time and the intervention groups are reported in Table 3. The main effect of time (*F*_1,181_=10.95; *P*=.001) was statistically significant (H1) whereas the interaction between the intervention groups and time was not (H2). The results pertaining to H3, H4, and H5 suggested no effect of the exposure to a specific module during the course of the intervention. Sensitivity analysis using a robust approach confirmed the same results (Multimedia Appendix 4). In addition, as a part of a further sensitivity analysis, the main hypotheses were tested without applying any missing value imputation algorithm. The results are available in Multimedia Appendix 4 and confirm the time effect and the null findings for the interaction effect.

**Table 3:** Main effects and interactions among modules on hand hygiene behavior at key times (N=232).

H^a^, outcome, and factor				Participants, n (%)		Parametric ANOVA				
						*F* test (*df*)		*P* value		Partial eta–squared^b^ (95% CI)
**H1 and H2**				190 (81.9)						
	**Hand hygiene**									
		Group^c^			0.33 (8)		.95		0.01 (0.00-1.00)	
		Time (T1-T3)			*10.95* ^d^ *(1)*		*.001*		*0.06 (0.01-1.00)*	
		Time × group			1.19 (8)		.31		0.05 (0.00-1.00)	
**H3**				190 (81.9)						
	**Hand hygiene**									
		Habit			1.25 (1)		.27		0.01 (0.00-1.00)	
		Time (T1-T3)			*10.87 (1)*		*.001*		*0.05 (0.01-1.00)*	
		Time × habit			1.07 (1)		.30		0.01 (0.00-1.00)	
**H4**				190 (81.9)						
	**Hand hygiene**									
		Motivation			0.00 (1)		.99		0.00 (0.00-1.00)	
		Time (T1-T3)			*10.86 (1)*		*.001*		*0.05 (0.01-1.00)*	
		Time × motivation			0.94 (1)		.33		0.00 (0.00-1.00)	
**H5**				190 (81.9)						
	**Hand hygiene**									
		Social			0.75 (1)		.39		0.00 (0.00-1.00)	
		Time (T1-T3)			*10.83 (1)*		*.001*		*0.05 (0.01-1.00)*	
		Time × social			0.41 (1)		.52		0.00 (0.00-1.00)	
**H6**				148 (63.8)						
	**Engagement**									
		Group (T3)			*2.19 (8)*		*.03*		*0.11 (0.01-1.00)*	
**H7**				148 (63.8)						
	**Usability**									
		Group (T3)			*2.46 (8)*		*.02*		*0.12 (0.01-1.00)*	
**H8**				148 (63.8)						
	**Satisfaction**									
		Group (T3)			1.46 (8)		.18		0.11 (0.00-1.00)	

^a^H: hypothesis.

^b^Partial eta–squared corresponds to the proportion of variance explained by a variable that is not explained by other variables.

^c^Group: intervention group.

^d^Italicized values indicate significance.

### Second Optimization Criterion: Participant Engagement, Usability, and Satisfaction

#### Quantitative Analysis

The effects of the intervention group on engagement, usability, and satisfaction are shown in Table 3. The results of the parametric ANOVA suggested that the self-reported measures of engagement (*F*_8,139_=2.19; *P*=.03) and usability (*F*_8,139_=2.46; *P*=.02) differed across the 9 intervention groups. Nonparametric ANOVA with Kruskal-Wallis test showed significant differences across the intervention groups only for usability (*χ*^2^_8_=16.1; *P*=.04). However, both parametric and nonparametric post hoc comparisons with Bonferroni adjustment indicated no mean score differences in engagement and usability between any pair of intervention groups.

#### Qualitative Analysis

##### Overview

Across 9 interviews, 7 themes emerged in relation to the research question (refer to Multimedia Appendix 5 for a summary of the themes and for additional extracts illustrating each theme). The themes were named “user experience and app functionality,” “importance of guidance,” “variety and timeliness of the task load,” “reasons for participation,” “change in awareness of hand hygiene and its implications,” “social interaction,” and “personal relevance.” In addition, the following 2 subthemes were identified as a part of the “social interaction” theme: “personal communication and connectedness” and “social comparison.”

##### User Experience and App Functionality

The first theme that emerged concerned the user experience with the general aesthetics and functionalities of the app. Overall, satisfaction with the intuitive and simple handling of the app was high. The participants considered the usability to be pleasant. Regarding the app aesthetics, some participants were very satisfied with the simplicity of the layout; however, the majority would have preferred more visual structures:

What I liked in particular? Actually, how things were presented. Just the simplicity—all in all it was very simple.

P7, habit-habit, moderate Adherence

Another point on which most participants agreed was that certain features of the app showed technical flaws, which negatively affected their motivation:

So, when this annoying technical problem occurred—if you were to draw a curve now, it [my motivation] went up quite steadily at the beginning, and then slowly decreased due to this technical problem, and then when it was resolved it [my motivation] got back up again.

P5, motivation-social, high adherence

##### Importance of Guidance

Throughout the interviews, the participants regularly highlighted the importance of receiving guidance within the app. Specifically, they mentioned the importance of clarity and meaning regarding the tasks that the app asked them to do:

I also thought it was nice that you kind of knew in the morning “ah today is a day with a big survey,” so that you could already plan “okay, today there are maybe a little bit more push messages coming in and I have to pay a little bit more attention.”

P2, habit-habit, high adherence

The importance of guidance was also manifested as the need for a better overview of the participants’ journey during the study. For instance, some participants would have liked more background information about the study to better understand the timeline or the reasons behind receiving certain tasks:

And otherwise maybe somehow a little bit more background information about what—why am I being asked these questions, so that I can see even more behind this algorithm and behind this concept and then it would become clearer to me why the same questions keep coming. So, a little bit, so even more background knowledge.

P3, social-habit, low adherence

Although guidance was acknowledged as important, too much direction was also perceived as overwhelming, for example, very frequent push notifications:

Was that now at 10 o’clock, at 12 o’clock or at 2 o’clock, I do not remember any more in which intervals the push messages came in. At the end, I no longer knew at what point I had I received the last push notification—there, I lost overview.

P2, habit-habit, high adherence

##### Variety and Timeliness of the Task Load

Variety in daily engagement with the content of the app emerged as a central topic in the interviews. A few participants were satisfied with the degree of variety in the task load and the timing of the content offered by the intervention. However, most participants wished for substantially more variety in the task load and timing, particularly toward the end of the intervention:

Sometimes, it was just quiet, nothing happened. But later, once again it came “today something is happening,” yes, I liked that.

P2, habit-habit, high adherence

Towards the end, when there were fewer and fewer exercises, I found it almost a bit boring.

P7, habit-habit, moderate adherence

##### Reasons for Participation

In most interviews, the participants mentioned their initial reasons for participating in the study. One of the most frequently reported motives was curiosity and an interest in learning something new:

I thought, “yeah, sure, I can wash my hands. But do I know everything when they do a study? I could still learn something at the end, I’m not omniscient.” And that's actually what mainly motivated me, this openness, I'm curious to see what else there is to learn.

P1, habit-habit, moderate adherence

The participants also elaborated on why they kept using the app. One of the cited motives was the perceived obligation to complete the study:

Well, it [my motivation] certainly did not increase, it was more a matter of persevering—in the sense of whoever says A must also say B. It was said that you could drop out at any time, but still.

P9, motivation-motivation, low adherence

##### Change in Awareness of Hand Hygiene and Its Implications

A further theme was represented by the increase in participants’ hand hygiene awareness owing to the use of the app. The change in awareness seemed to have been generated by the fact that participants paid more attention to the self-monitoring of the target behavior:

That was simply my observation of my reaction then—you observe yourself during these four weeks incredibly—I do not know if you have also heard this from other people, but you start watching yourself.

P5, motivation-social, high adherence

The change in awareness generated a positive loop that led to an increase in the frequency of hand hygiene behavior together with a shift in perception of the issue of hand hygiene and its implications:

I certainly washed my hands more than I had before. And therefore, I have the feeling that I have certainly benefitted from it [the intervention].

P4, habit-motivation, high adherence

##### Social Interaction

The theme of social interaction came up several times during most interviews. Two subthemes define this main theme according to the different social aspects that came to light during the interviews: personal communication and connectedness and social comparison.

##### Subtheme 1: Personal Communication and Connectedness

Some participants particularly appreciated that the app communicated with them in a personal manner. This led to a feeling of authenticity; therefore, these participants no longer had the impression of interacting with a machine when using the app:

You can say that there is someone behind it. I never felt alone, it was not a one-way kind of communication. I always knew that behind these tasks was indeed a computer, but I still felt connected in a way.

P2, habit-habit, high adherence

By contrast, other participants would have preferred an even more human-centered mode of delivery of the app content, for example, receiving direct motivational support from other humans:

Maybe, despite everything, a video or something like that—or actually, as is often the case nowadays: a small video with other participants who motivate you. Because reading statistics and news is something else than when someone speaks directly to you.

P8, social-habit, low adherence

Some participants described having developed a feeling of connectedness with other app users over time. This led to a sense of community, which made them feel supported:

And then I think I had to answer this question three times. And at the end, I think that was at the final question, I thought “yes, I think it is cool that they are taking part, I do not know them, but I think it is cool that they are taking part, and I feel connected to them.”

P2, habit-habit, high adherence

##### Subtheme 2: Social Comparison

The participants who were exposed to the social module shared different opinions regarding the opportunity to compare their behavior with that of other participants, which was a feature of the social module only. Indeed, although some participants expressed avoidance of social comparison and fatigue with the competition it created for them, others were pleased about the comparison with other users:

For me, personally, it was too much with the community and otherwise, because others cannot motivate me. Whether someone somehow achieved 100% or 50%, that is actually relatively indifferent to me. And it does not encourage me to become more or less active or whatever.

P8, social-habit, low adherence

##### Wish for Personalization

An issue raised by almost every participant was the lack of personal relevance that the list of key moments for hand hygiene entailed. Being regularly asked about key situations that never occurred for them (eg, not having children or not wearing contact lenses) led to a decrease in motivation to fill out the hand hygiene diaries:

Things are asked again and again, which do not concern you at all. This leads to a decrease in motivation. Now, I have to spend five minutes filling out the form again, even though it does not apply.

P3, social-habit, low adherence

The desire to personalize the app also came up in relation to other intervention content, such as the number of push notifications:

But maybe in the beginning you should be able to specify “I would rather have a little more [push notifications] or a little less.” But what I have received, however, has been right for me.

P2, habit-habit, high adherence

## Discussion

### Principal Findings

As part of a MOST to develop and test a smartphone-based hand hygiene intervention during the COVID-19 pandemic, this intervention optimization parallel randomized trial aimed to identify the best combination of intervention modules to be included in the subsequent evaluation phase of Soapp. The results from the main analyses confirmed that the participants who participated in the study increased the frequency of correct hand hygiene at key times over time (H1). However, the intervention groups did not differ in their effects on correct hand hygiene at key times (H2). Similarly, the exposure to specific modules was not associated with increased hand hygiene over time (H3, H4, and H5). Taken together, the findings related to the first optimization criterion suggested a promising increase in hand hygiene during the intervention period but did not provide scientific evidence to support the preference of one version of Soapp or a specific module over the others. Similarly, the quantitative results from the second optimization criterion (H6, H7, and H8) did not show any differences in engagement, usability, and satisfaction among the 9 intervention modules at T3.

By contrast, the qualitative results revealed what characteristics and features of Soapp the participants perceived as supportive or, conversely, detrimental in terms of engagement, usability, and satisfaction. The finding that the aesthetics and design of the app are important for participants to better enjoy their interaction with Soapp is in line with a previous study on health-related behavior change [29]. The participants expressed the desire for an app that is simple to use, intuitive, and not cognitively demanding and that allows a smooth use of its functionalities. Such fundamental characteristics are deemed to guarantee satisfactory and engaging user experiences with Soapp. A second relevant aspect raised by the interviewed participants is the desire to receive clear guidance about the tasks that the app proposes and the rationale behind them. The participants also appreciated when they received (1) information regarding the behavior change intervention they committed to and (2) suggestions (eg, tips and problem-solving strategies) on how to adhere to correct hand hygiene. However, to prevent declining engagement, the delivery of guiding content should be balanced and not overwhelming (eg, push notifications). These findings are in line with those found in a previous systematic review and empirical research on engagement with digital behavior change interventions [30-32]. A further topic is variety in the daily interactions with the app and the proposed tasks and activities. A task load that varies daily (ie, days with more tasks and days with fewer tasks) seems to be important for sustaining engagement with Soapp. In addition, the regular provision of content over the course of the intervention was considered an important aspect of the app that might require some improvements. This aspect is of particular relevance, as the receipt of an optimal dose of engagement may increase the effectiveness of digital interventions [30]. Another theme that emerged during the interviews concerned the reasons that led the participants to join and remain engaged with the study. Although curiosity and interest to learn new things were important in triggering initial engagement, perceived obligation was a reason to maintain engagement over time. This result provides further support to participants’ demand for a better distributed workload and content over the course of the use of Soapp. On the content side, as in a previous study about adults’ perspectives on health behavior change apps [33], the participants appreciated those features that foster an increased sense of awareness around the target health-related behavior (ie, hand hygiene) and the resulting benefits. These results are in line with recent research conducted during the COVID-19 pandemic suggesting that self-monitoring is positively associated with hand washing [34]. Interestingly, the participants reported that the awareness formed mostly because of filling out the hand hygiene diaries that were included in the study as assessment tools and not as behavior change techniques (ie, self-monitoring). This aspect underlines how assessment tools and intervention strategies were not distinguished from one another by the participants but were perceived as part of the same user experience. A further theme that was at the center of the participants’ comments regarded social interaction. Consistent with previous findings [30-32,35], features supporting a sense of relatedness owing to both a human-centered communication style (ie, tone of voice) and a feeling of connectedness were considered necessary to create social commitment and, ultimately, for engagement and satisfactory interactions with Soapp. Such a sense of relatedness was generated by the general user experience provided by the app (eg, communication style) and was not related to the features delivered by the social norm module. By contrast, in line with previous findings regarding health-related digital interventions [29,31-33,35], a dual perspective emerged in relation to the features that purposefully provided opportunities for social comparison and were part of the social norms module. Indeed, although some participants expressed avoidance of social comparison because they considered their behavior change journey as a personal dimension of their life, others were pleased about the comparison with other users. Therefore, social comparison features can be seen as a 2-edged sword for engagement, as the preference for such features is expected to vary across individuals. Eventually, the participants believed that receiving more personally relevant content would strengthen their engagement with Soapp. Such comments were partially generated by the participants’ experiences in filling out hand hygiene diaries that refer to key times that are not relevant to them.

### Implications for the Evaluation Phase of Soapp

Owing to the null findings of the first optimization criterion, we were not able to identify the best intervention group based on the quantitative analysis of the primary outcome. Similarly, no between-group differences emerged in relation to the second optimization criterion (ie, engagement, usability, and satisfaction). Therefore, we relied on the results of the thematic analysis to derive implications for the evaluation phase of Soapp. The resulting intervention design decisions based on this optimization study are summarized in Table 4.

**Table 4:** Intervention design recommendations for the evaluation phase of Soapp.

Recommendation	Rationale
The social module is excluded from the next evaluation phase.	Habit and motivation modules seem best suited to leverage some of the themes that emerged during the thematic analysis. For instance, themes such as change in awareness and guidance can be better supported by the app features that characterize these modules (ie, action planning tasks, self-monitoring, opportunity to schedule custom reminder, and video on health implication). In addition, the social module might be detrimental for engagement, as it embeds social comparison features, which were perceived as counterproductive by some users.
A parallel delivery of modules is preferrable over a sequential one.	The specific sequence of intervention modules (ie, habit-motivation vs motivation-habit) was not associated with differences in hand hygiene. Therefore, according to the participants’ needs identified as part of the theme “variety and timeliness of the task load,” a parallel delivery of the selected intervention modules is preferable.
Define a more even distribution of the intervention content and notifications over the course of the study.	A parallel delivery of the modules would allow to distribute each module’s content and tasks over 32 days instead of 16 days, as done during the optimization phase. Therefore, there is more flexibility to define the timeline of the intervention with the aim of balancing the daily task load and, ultimately, guaranteeing a more suited dose of content over the course of the intervention.

### Limitations

This study is not without limitations. A main weakness is the sample size achieved for testing the main hypotheses. Indeed, an a posteriori achieved power of 0.44 (n=190, 81.9%; α=.05; partial η^2^=0.01) suggests that the probability of detecting a true effect of the intervention groups was lower than the recommended standard (ie, 0.80). Different factors contributed to the collection of data from a limited sample of 190 participants. First, we stopped recruitment 5 months after the start of the study, although the target sample size was not achieved. As specified in the study protocol, the criterion of discontinuing the enrollment of participants after 5 months was based on the constraints of the project timeline [16]. This resulted in a sample of 232 randomized participants. A second reason is the dropouts between randomization and T1 assessment. The T1 assessment was scheduled for the day after randomization; however, some (42/232, 18.1%) participants who had been randomized did not complete it. Therefore, they were excluded from the main analyses.

A further limitation that affected the analysis of the primary outcome was attrition. Of the 190 participants who filled out the T1 diary, 118 (62.1%) completed the diary at T3, leading to 38% and 49% attrition compared with the T1 and randomization figures, respectively. The attrition rate was higher than the estimated rate (ie, 20%). To account for potential differences between dropouts and retainers, we conducted a dropout analysis to investigate whether they differed in regard to key variables such as age, sex, hand hygiene, and intention to increase hand hygiene behavior. Because no significant differences emerged, we considered the participants who completed the study to be representative of our target population (ie, adults interested in using an app to improve hand hygiene behavior). A possible explanation for attrition could be the possibility that the longitudinal study design with 5 diary days and further quasidaily tasks might have generated an interaction fatigue. In addition, the pandemic trajectory during the enrollment period flattened in Switzerland, which may have made hand hygiene less of a priority for potential participants. To overcome this issue and in line with the ITT approach, we used the last observation carried forward method to replace the missing observations in the T3 diary with the latest available diary assessment. However, it should be noted that this method is based on the assumption that behavior is stable and, therefore, might have introduced bias.

Furthermore, the recruited sample was characterized by a high prevalence of women (ie, 139/190, 73.2%). This imbalance was in line with previous research on hand hygiene during the COVID-19 pandemic [11]. A plausible explanation for this gender imbalance might be that during the COVID-19 pandemic, women tended to show higher levels of worry and fear of the pandemic and were keener to adopt protective behaviors such as hand hygiene [11,36].

Finally, the self-reported measurement of hand hygiene may be biased. The use of an electronic diary to measure hand hygiene behavior at key times should have had limited retrospective bias. However, social desirability cannot be disregarded. In addition, thematic analysis indicated that the diary may have worked as an unintentional behavior change technique (ie, self-monitoring).

### Conclusions

This study described the optimization phase of Soapp, a smartphone app for promoting hand hygiene in the context of the COVID-19 pandemic. By leveraging digital technologies and MOST, we addressed the call raised by public health experts for developing evidence-based behavior change interventions that are designed and optimized to be effective in a pandemic context [5]. In this regard, we provided support for the feasibility and effectiveness of digital interventions promoting hand hygiene behavior during an ongoing pandemic. This aspect is extremely relevant because digital interventions do not require personal contact and can be integrated into the daily lives of an unlimited number of people. Furthermore, our findings contributed to filling an existing research gap and improving the scientific knowledge on the most effective behavior change strategies to promote hand hygiene during a pandemic. Ultimately, Soapp represents a promising ready-to-go digital tool to be used in cases of future pandemics.

## Appendix

Multimedia Appendix 1 Hand hygiene items and secondary hypothesis testing.

Multimedia Appendix 2 CONSORT (Consolidated Standards of Reporting Trials) checklist.

Multimedia Appendix 3 Qualitative interview guide.

Multimedia Appendix 4 Supplement to quantitative results.

Multimedia Appendix 5 Summary and quotes from the thematic analysis.

## Abbreviations

CONSORT: Consolidated Standards of Reporting Trials
DBCI: digital behavior change interventions
H: hypothesis
ITT: intention-to-treat
MOST: Multiphase Optimization Strategy
REDCap: Research Electronic Data Capture
T1: baseline
T3: follow-up
ZUF: Fragebogen zur Messung der Patientenzufriedenheit

## References

[1] Rabie T, Curtis V (2006). Handwashing and risk of respiratory infections: a quantitative systematic review. Trop Med Int Health.

[2] Aiello AE, Coulborn RM, Perez V, Larson EL (2008). Effect of hand hygiene on infectious disease risk in the community setting: a meta-analysis. Am J Public Health.

[3] Beale S, Johnson AM, Zambon M, Hayward AC, Fragaszy EB, Flu Watch Group (2021). Hand hygiene practices and the risk of human coronavirus infections in a UK community cohort. Wellcome Open Res.

[4] Lee JK, Bullen C, Ben Amor Y, Bush SR, Colombo F, Gaviria A, Karim SS, Kim B, Lavis JN, Lazarus JV, Lo YC, Michie SF, Norheim OF, Oh J, Reddy KS, Rostila M, Sáenz R, Smith LD, Thwaites JW, Were MK, Xue L, (The Lancet COVID-19 Commission Task Force for Public Health Measures to Suppress the Pandemic) (2021). Institutional and behaviour-change interventions to support COVID-19 public health measures: a review by the Lancet Commission Task Force on public health measures to suppress the pandemic. Int Health.

[5] Michie S, West R (2020). Behavioural, environmental, social, and systems interventions against COVID-19. BMJ.

[6] Perski O, Szinay D, Corker E, Shahab L, West R, Michie S (2022). Interventions to increase personal protective behaviours to limit the spread of respiratory viruses: a rapid evidence review and meta-analysis. Br J Health Psychol.

[7] Ainsworth B, Miller S, Denison-Day J, Stuart B, Groot J, Rice C, Bostock J, Hu XY, Morton K, Towler L, Moore M, Willcox M, Chadborn T, Gold N, Amlôt R, Little P, Yardley L (2021). Infection control behavior at home during the COVID-19 pandemic: observational study of a web-based behavioral intervention (germ defence). J Med Internet Res.

[8] Wise T, Zbozinek TD, Michelini G, Hagan CC, Mobbs D (2020). Changes in risk perception and self-reported protective behaviour during the first week of the COVID-19 pandemic in the United States. R Soc Open Sci.

[9] Moussaoui LS, Ofosu ND, Desrichard O (2020). Social psychological correlates of protective behaviours in the COVID-19 outbreak: evidence and recommendations from a nationally representative sample. Appl Psychol Health Well Being.

[10] Lippke S, Keller FM, Derksen C, Kötting L, Dahmen A (2022). Hygiene behaviors and SARS-CoV-2-preventive behaviors in the face of the COVID-19 pandemic: self-reported compliance and associations with fear, SARS-CoV-2 risk, and mental health in a general population vs. a psychosomatic patients sample in Germany. Hygiene.

[11] Szczuka Z, Abraham C, Baban A, Brooks S, Cipolletta S, Danso E, Dombrowski SU, Gan Y, Gaspar T, de Matos MG, Griva K, Jongenelis M, Keller J, Knoll N, Ma J, Miah MA, Morgan K, Peraud W, Quintard B, Shah V, Schenkel K, Scholz U, Schwarzer R, Siwa M, Szymanski K, Taut D, Tomaino SC, Vilchinsky N, Wolf H, Luszczynska A (2021). The trajectory of COVID-19 pandemic and handwashing adherence: findings from 14 countries. BMC Public Health.

[12] Michie S, Richardson M, Johnston M, Abraham C, Francis J, Hardeman W, Eccles MP, Cane J, Wood CE (2013). The behavior change technique taxonomy (v1) of 93 hierarchically clustered techniques: building an international consensus for the reporting of behavior change interventions. Ann Behav Med.

[13] McKay FH, Wright A, Shill J, Stephens H, Uccellini M (2019). Using health and well-being apps for behavior change: a systematic search and rating of apps. JMIR Mhealth Uhealth.

[14] White S, Thorseth AH, Dreibelbis R, Curtis V (2020). The determinants of handwashing behaviour in domestic settings: an integrative systematic review. Int J Hyg Environ Health.

[15] Collins LM, Nahum-Shani I, Almirall D (2014). Optimization of behavioral dynamic treatment regimens based on the sequential, multiple assignment, randomized trial (SMART). Clin Trials.

[16] Amrein MA, Ruschetti GG, Baeder C, Bamert M, Inauen J (2022). Mobile intervention to promote correct hand hygiene at key times to prevent COVID-19 in the Swiss adult general population: study protocol of a multiphase optimisation strategy. BMJ Open.

[17] Schulz KF, Altman DG, Moher D, CONSORT Group (2010). CONSORT 2010 statement: updated guidelines for reporting parallel group randomised trials. BMC Med.

[18] Faul F, Erdfelder E, Buchner A, Lang AG (2009). Statistical power analyses using G*Power 3.1: tests for correlation and regression analyses. Behav Res Methods.

[19] Glaser BG, Strauss AL (1999). The Discovery of Grounded Theory: Strategies for Qualitative Research.

[20] Perski O, Blandford A, Garnett C, Crane D, West R, Michie S (2020). A self-report measure of engagement with digital behavior change interventions (DBCIs): development and psychometric evaluation of the "DBCI Engagement Scale". Transl Behav Med.

[21] Peres SC, Pham T, Phillips R (2013). Validation of the system usability scale (SUS): SUS in the wild. Proc Hum Factors Ergon Soc Annu Meet.

[22] Schmidt J, Lamprecht F, Wittmann WW (1989). Zufriedenheit mit der stationären Versorgung. Entwicklung eines Fragebogens und erste Validitätsuntersuchungen. Psychother Psychosom Med Psychol.

[23] Harris PA, Taylor R, Minor BL, Elliott V, Fernandez M, O'Neal L, McLeod L, Delacqua G, Delacqua F, Kirby J, Duda SN, REDCap Consortium (2019). The REDCap consortium: building an international community of software platform partners. J Biomed Inform.

[24] Contzen N, De Pasquale S, Mosler HJ (2015). Over-reporting in handwashing self-reports: potential explanatory factors and alternative measurements. PLoS One.

[25] McCoy CE (2017). Understanding the intention-to-treat principle in randomized controlled trials. West J Emerg Med.

[26] Garnett C, Oldham M, Angus C, Beard E, Burton R, Field M, Greaves F, Hickman M, Kaner E, Loebenberg G, Michie S, Munafò M, Pizzo E, Brown J (2021). Evaluating the effectiveness of the smartphone app, Drink Less, compared with the NHS alcohol advice webpage, for the reduction of alcohol consumption among hazardous and harmful adult drinkers in the UK at 6-month follow-up: protocol for a randomised controlled trial. Addiction.

[27] Baretta D, Inauen J (2022). SOAPP - Hand Hygiene promotion in the context of the COVID-19 pandemic. Open Science Framework.

[28] Braun V, Clarke V (2006). Using thematic analysis in psychology. Qual Res Psychol.

[29] Perski O, Blandford A, Ubhi HK, West R, Michie S (2017). Smokers' and drinkers' choice of smartphone applications and expectations of engagement: a think aloud and interview study. BMC Med Inform Decis Mak.

[30] Perski O, Blandford A, West R, Michie S (2017). Conceptualising engagement with digital behaviour change interventions: a systematic review using principles from critical interpretive synthesis. Transl Behav Med.

[31] Perski O, Baretta D, Blandford A, West R, Michie S (2018). Engagement features judged by excessive drinkers as most important to include in smartphone applications for alcohol reduction: a mixed-methods study. Digit Health.

[32] Baretta D, Perski O, Steca P (2019). Exploring users' experiences of the uptake and adoption of physical activity apps: longitudinal qualitative study. JMIR Mhealth Uhealth.

[33] Dennison L, Morrison L, Conway G, Yardley L (2013). Opportunities and challenges for smartphone applications in supporting health behavior change: qualitative study. J Med Internet Res.

[34] Keller J, Kwasnicka D, Wilhelm LO, Lorbeer N, Pauly T, Domke A, Knoll N, Fleig L (2022). Hand washing and related cognitions following a brief behavior change intervention during the COVID-19 pandemic: a pre-post analysis. Int J Behav Med.

[35] D'Addario M, Baretta D, Zanatta F, Greco A, Steca P (2020). Engagement features in physical activity smartphone apps: focus group study with sedentary people. JMIR Mhealth Uhealth.

[36] Bronfman N, Repetto P, Cordón P, Castañeda J, Cisternas P (2021). Gender differences on psychosocial factors affecting COVID-19 preventive behaviors. Sustainability.

