# Supplementary material for: Promoting Hand Hygiene During the COVID-19 Pandemic: Parallel Randomized Trial for the Optimization of the Soapp App
Source: JMIR Mhealth Uhealth. 2023 Feb 3;11:e43241. doi: 10.2196/43241 (PMC9938438; doi:10.2196/43241)
Supplement: Multimedia Appendix 1 [file mhealth_v11i1e43241_app1.docx]

**Multimedia Appendix 1**

**List of all key times when to perform correct hand hygiene according to the Swiss Federal Office of Public Health**

| **Item** | **Key times** | **Type** |
| --- | --- | --- |
| 1 | Before preparing the meal or before sitting down at the table | General |
| 2 | Before eating or before feeding the children | General |
| 3 | After blowing your nose, sneezing, or coughing | General |
| 4 | Every time you come home | General |
| 5 | After using public transport | General |
| 6 | After visiting sick people or after close contact with material from sick people or with their personal effects | General |
| 7 | Before inserting and removing the contact lenses | General |
| 8 | After taking off the mask | COVID-19 specific |
| 9 | After going to the toilet or accompanying a child to the toilet (including after changing diapers) | General |
| 10 | After handling waste | General |
| 11 | If you have dirty hands or if they are visibly dirty | General |
| 12 | After visiting public places | COVID-19 specific |
| 13 | After touching surfaces outside the home or money | COVID-19 specific |
| *Note.* Type = ‘General’ indicates key times that are recommended in general and before the COVID-19 pandemic occurred. | | |
|  | | |

**Secondary hypotheses^[[1]](#footnote-1)^**

**Hypothesis 3**

The intervention groups show a significant increase in incorrectly performed hand hygiene at key times after 4 weeks (T3) of intervention compared with baseline (T1).

**Measures:**

- Frequency of hand hygiene behaviour at key times, but not correctly.

*How many times they wash or disinfect their hands, but not correctly? The responses are: 0 = never, 1 = rarely, 2 = sometimes, 3 = often, 4 = always. Participants receive 5 questionnaires during a diary day. People attend a diary day after module 1 and after module 2. The frequencies across the 5-times daily diaries will be averaged to indicate the average frequency by which participants not-correctly performed hand hygiene at key times that day (scale ranging from 0 to 4).*

**Results:**

| **Outcome** | **Factor** | **N** | **Group means** | **F** | **df** | **p-value** | **Part Eta Sq^1^** | **95% CI^2^** |
| --- | --- | --- | --- | --- | --- | --- | --- | --- |
| Incorrectly performed hand hygiene | Time (T1-T3) | 190 | [2.29; 2.09] | 5.86 | 1 | <.05 | 0.03 | [0.00, 1.00] |
| *Note.* ^1^Part Eta Sq = Partial Eta Squared corresponds to the proportion of variance that a variable explains that is not explained by other variables; ^2^CI = Confidence Intervals; in bold significant results. | | | | | | | | |

**Box plot for significant results**


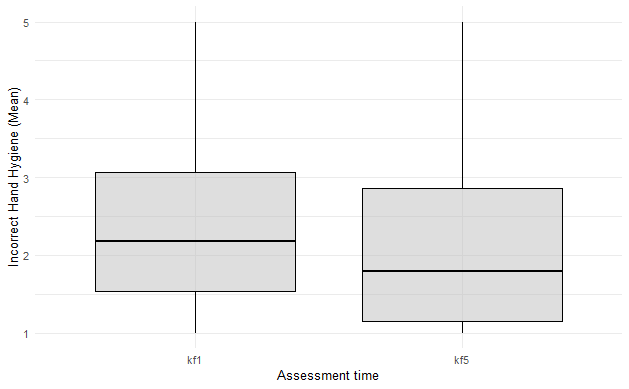


*Note.* The black horizontal line is the median value.

**Hypothesis 4**

The groups attending a motivation module show a significant increase in (a) intention, (b) self-efficacy, (c) outcome expectancies, and (d) attitude from the start of the intervention to the measures 2 weeks (T2) and 4 weeks (T3) into the intervention compared with baseline (T1).

**Measures:**

- Self-reported behavioral intention

*One item: "To what extent do you intend to correctly perform your hand hygiene behavior at key times?" The response options ranged from 1 "not at all" to 6 "very strongly." Higher scores indicate stronger intention.*

- Mean score of eight self-reported outcome-expectancies items

*Eight items such as "If I do not perform correct hand hygiene behavior at key times, then I stay rather healthy" The response options ranged from 1 "not at all" to 6 "very strongly." Higher scores indicate higher outcome-expectancies.*

- Mean score of six self-reported attitude items [ Time Frame: Day 17 (main survey T2) and day 34 (main survey T3) ]

*Six items such as "Correct hand hygiene behavior at key times is…" response range from pleasant to unpleasant. Higher scores indicate higher attitude.*

**Results**

| **Outcome** | **Reliability^1^** | **Within Factor** | **Group means** | **N** | **F** | **df** | **p-value** | **Part Eta Sq^2^** | **95% CI^3^** |
| --- | --- | --- | --- | --- | --- | --- | --- | --- | --- |
| Intention | - | Time (T1-T2) | [5.00; 5.00] | 47 | .00 | 1 | 1 | 0.00 | [0.00, 1.00] |
|  | - | Time (T2-T3) | [5.00; 4.95] | 37 | 1.00 | 1 | .324 | 0.03 | [0.00, 1.00] |
|  | - | Time (T1-T3) | [5.08; 5.11] | 69 | .15 | 1 | .698 | 0.00 | [0.00, 1.00] |
| Self-efficacy | .92 | **Time (T1-T2)** | [4.08; 4.42] | **46** | **8.40** | **1** | **<.01** | **.16** | **[0.03, 1.00]** |
|  | .91 | **Time (T2-T3)** | [4.43; 4.72] | **37** | **17.59** | **1** | **<.001** | **.33** | **[0.13, 1.00]** |
|  | .92 | **Time (T1-T3)** | [4.34; 4.67] | **75** | **22.00** | **1** | **<.001** | **.23** | **[0.10, 1.00]** |
| Outcome expectancies | .67 | Time (T1-T2) | [4.48; 4.34] | 46 | 3.76 | 1 | .058 | .08 | [0.00, 1.00] |
|  | .78 | Time (T2-T3) | [4.50; 4.59] | 37 | 1.83 | 1 | .148 | .05 | [0.00, 1.00] |
|  | .78 | Time (T1-T3) | [4.62; 4.61] | 75 | 5.71 | 1 | .981 | .00 | [0.00, 1.00] |
| Attitudes | .90 | Time (T1-T2) | [4.61; 4.77] | 45 | .96 | 1 | .333 | .02 | [0.00, 1.00] |
|  | .88 | Time (T2-T3) | [4.79; 4.71] | 37 | .88 | 1 | .354 | .02 | [0.00, 1.00] |
|  | .84 | Time (T1-T3) | [4.76; 4.97] | 74 | 3.19 | 1 | .078 | .04 | [0.00, 1.00] |
| *Note*. ^1^Reliability is assessed using Cronbach’s Alpha – when alpha is not displayed is because the scale is represented by a single item; ^2^Part Eta Sq = Partial Eta Squared corresponds to the proportion of variance that a variable explains that is not explained by other variables; ^3^CI = Confidence Intervals; in bold significant results. | | | | | | | | | |

**Box plots**


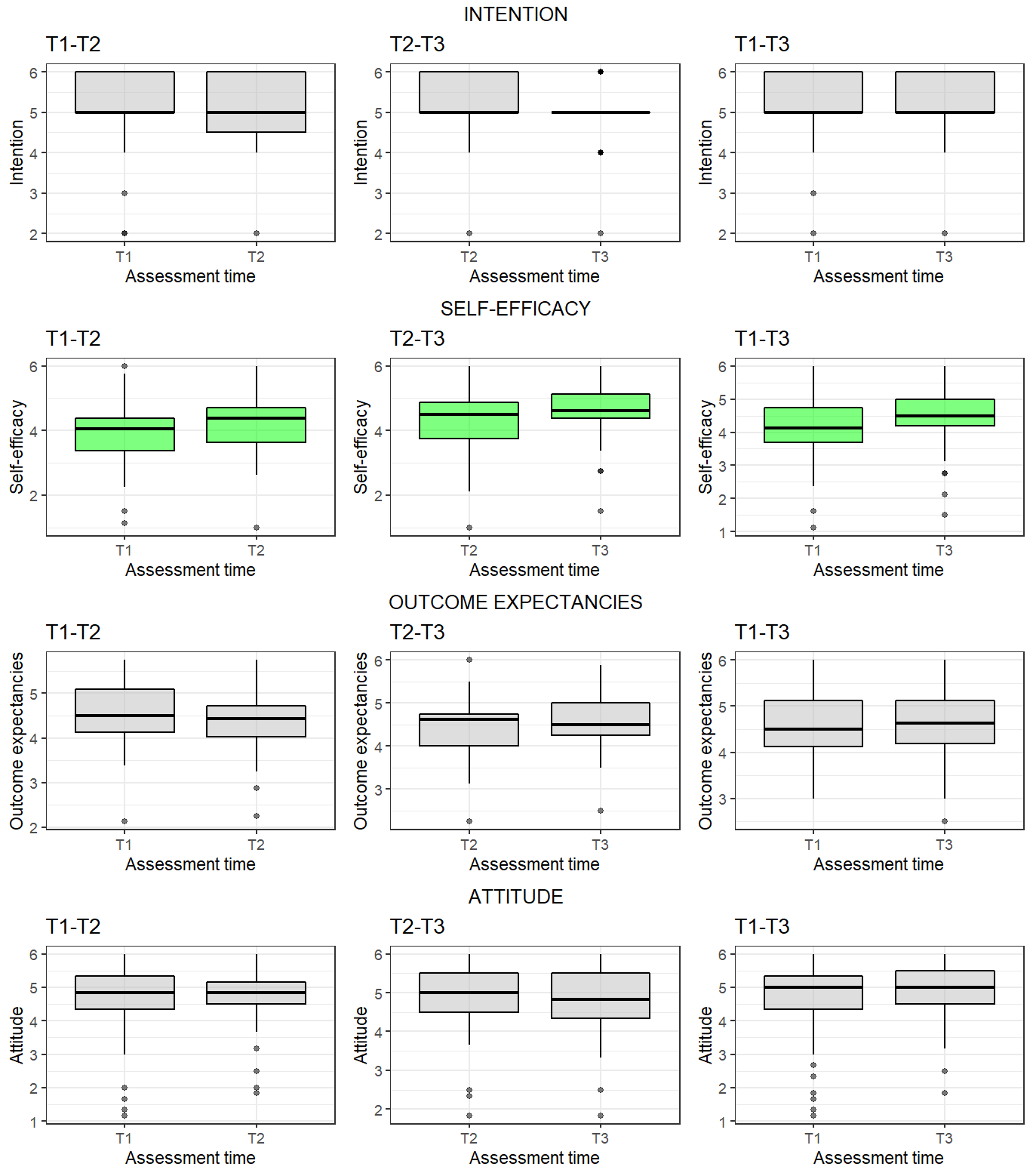


*Note.* In green significant differences. The black horizontal line is the median value.

**Hypothesis 5**

The groups attending a habit module show a significant increase in (a) habit strength, (b) action control, and (c) planning from the start of the intervention to the measures 2 weeks (T2) and 4 weeks (T3) into the intervention compared with baseline (T1).

**Measures:**

- Mean score of four self-reported coping planning items

*Four items such as: "I have made a detailed plan about how I can perform correct hand hygiene behavior at key times, when soap and water are not available' The response options ranged from 1 "not at all" to 6 "very strongly." Higher scores indicate higher coping planning.*

- Mean score of three self-reported action planning items [ Time Frame: Day 17 (main survey T2) and day 34 (main survey T3) ]

*Three items such as: "I have made a detailed plan about when I am going to wash and disinfect may hands." The response options ranged from 1 "not at all" to 6 "very strongly." Higher scores indicate higher action planning.*

- Habit strength assessed with the self-report habit index (SRHI) [ Time Frame: Day 17 (main survey T2) and day 34 (main survey T3) ]

*Self-Report Habit Index (SRHI) with four times such as: "Correct hand hygiene behavior at key times is something that I do automatically." The response options ranged from 1 "strongly disagree" to 6 "strongly agree"*

- Mean score of three self-reported action control items [ Time Frame: Day 17 (main survey T2) and day 34 (main survey T3) ]

*Three items such as "During the last two weeks I have constantly monitored myself whether I washed or disinfected my hands according to my plans" The response options ranged from 1 "not at all" to 6 "very strongly." With the additional item option "During the last two weeks, I did not intend to perform correct hand hygiene behavior at key times". Higher scores indicate higher action control.*

**Results**

| **Outcome** | **Reliability^1^** | **Within Factor** | **Group means** | **N** | **F** | **df** | **p-value** | **Part Eta Sq^2^** | **95% CI^3^** |
| --- | --- | --- | --- | --- | --- | --- | --- | --- | --- |
| Habit | .89 | Time (T1-T2) | [5.15; 4.93] | 52 | 3.42 | 1 | .070 | .06 | [0.00, 1.00] |
|  | .93 | Time (T2-T3) | [4.96; 5.02] | 48 | .11 | 1 | .736 | .00 | [0.00, 1.00] |
|  | .94 | Time (T1-T3) | [5.10; 5.07] | 85 | .09 | 1 | .762 | .00 | [0.00, 1.00] |
| Action Planning | .88 | **Time (T1-T2)** | [4.53; 4.84] | **52** | **7.20** | **1** | **<.01** | **.12** | **[0.02, 1.00]** |
|  | .85 | Time (T2-T3) | [4.67; 4.80] | 48 | 1.68 | 1 | .202 | .03 | [0.00, 1.00] |
|  | .91 | **Time (T1-T3)** | [4.45; 4.77] | **85** | **7.79** | **1** | **<.01** | **.08** | **[0.01, 1.00]** |
| Coping Planning | .87 | **Time (T1-T2)** | [4.17; 4.65] | **52** | **13.33** | **1** | **<.001** | **.21** | **[0.07, 1.00]** |
|  | .81 | **Time (T2-T3)** | [4.26; 4.63] | **48** | **5.83** | **1** | **<.05** | **.03** | **[0.00, 1.00]** |
|  | .89 | **Time (T1-T3)** | [4.03; 4.80] | **85** | **39.90** | **1** | **<.001** | **.32** | **[0.19, 1.00]** |
| Action Control | .90 | **Time (T1-T2)** | [4.34; 4.81] | **41** | **10.00** | **1** | **<.01** | **.20** | **[0.05, 1.00]** |
|  | .87 | **Time (T2-T3)** | [4.26; 4.63] | **48** | **5.83** | **1** | **<.05** | **.11** | **[0.01, 1.00]** |
|  | .86 | **Time (T1-T3)** | [4.58; 5.15] | **67** | **17.58** | **1** | **<.001** | **.21** | **[0.08, 1.00]** |
| *Note*. ^1^Reliability is assessed using Cronbach’s Alpha; ^2^Part Eta Sq = Partial Eta Squared corresponds to the proportion of variance that a variable explains that is not explained by other variables; ^3^CI = Confidence Intervals; in bold significant results. | | | | | | | | | |

**Box plots**


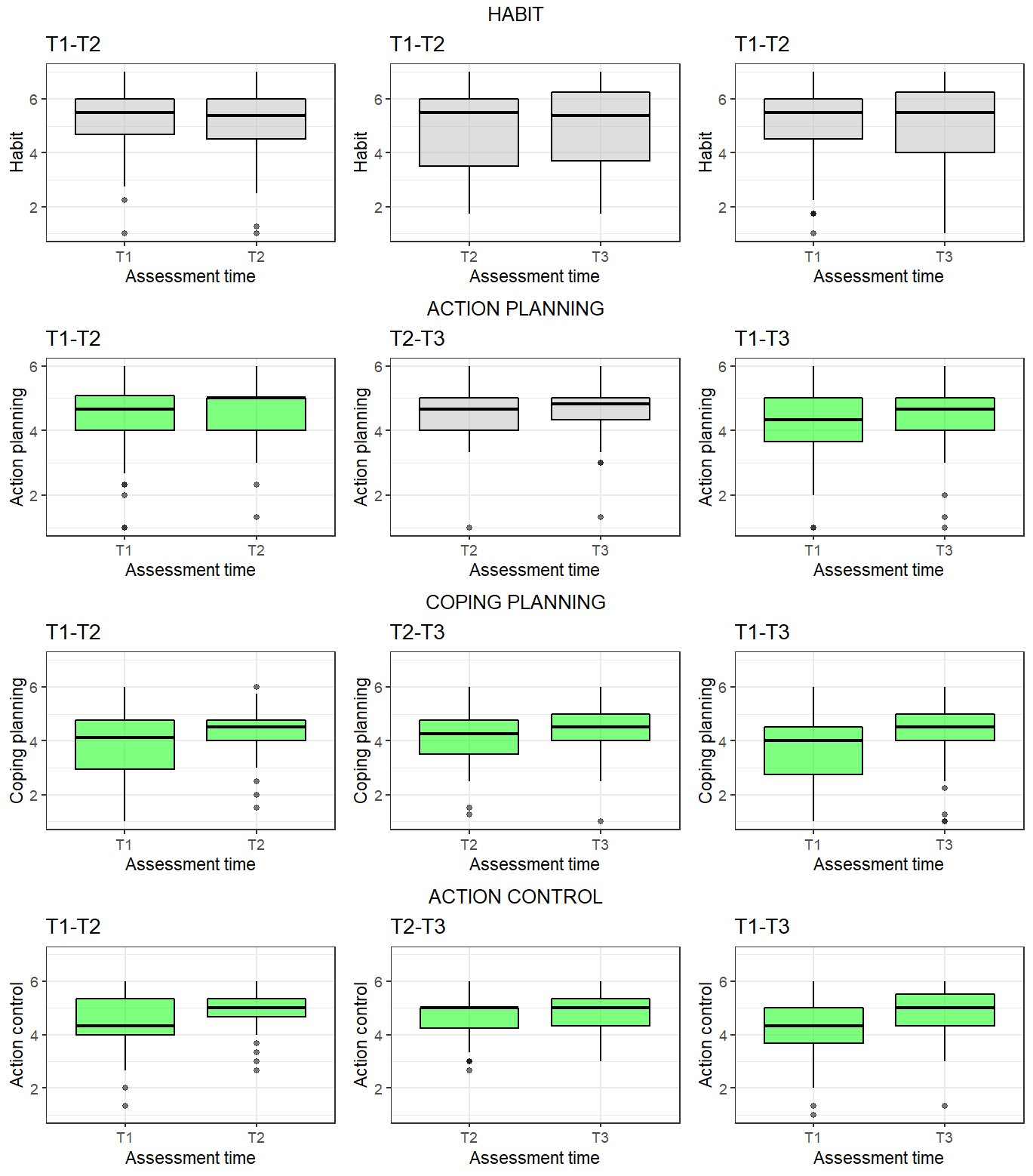


*Note.* In green significant differences. The black horizontal line is the median value.

**Hypothesis 6**

The groups attending a social norms module show a significant increase in (a) injunctive norms and (b) descriptive norms from the start of the intervention to the measures 2 weeks (T2) and 4 weeks (T3) into the intervention compared with baseline (T1).

**Measures:**

- Mean score of three self-reported injunctive norm items [ Time Frame: Day 17 (main survey T2) and day 34 (main survey T3) ]

*Three items such as "To what extent do you think that most people who are important to me approve correct hand hygiene behavior at key times? The response options ranged from 1 "not at all" to 6 "very strongly." Higher scores indicate higher injunctive norms.*

- Mean score of two self-reported descriptive norm items [ Time Frame: Day 17 (main survey T2) and day 34 (main survey T3) ]

*Two times such as "To what extent do you think that most people who are important to me perform correct hand hygiene behavior at key times? The response options ranged from 1 "not at all" to 6 "very strongly." Higher scores indicate higher descriptive norms.*

**Results**

| **Outcome** | **Reliability^1^** | **Within Factor** | **Group means** | **N** | **F** | **df** | **p-value** | **Part Eta Sq^2^** | **95% CI^3^** |
| --- | --- | --- | --- | --- | --- | --- | --- | --- | --- |
| Descriptive norms | .47 | Time (T1-T2) | [3.78; 3.81] | 52 | 5.56 | 1 | .815 | .00 | [0.00, 1.00] |
|  | .32 | Time (T2-T3) | [3.80; 3.98] | 47 | 2.13 | 1 | .151 | .04 | [0.00, 1.00] |
|  | .60 | Time (T1-T3) | [3.74; 3.92] | 85 | 3.49 | 1 | .065 | .04 | [0.00, 1.00] |
| Injunctive norms | .61 | Time (T1-T2) | [4.50; 4.55] | 52 | .22 | 1 | .637 | .00 | [0.00, 1.00] |
|  | .62 | Time (T2-T3) | [4.74; 4.77] | 47 | .07 | 1 | .797 | .00 | [0.00, 1.00] |
|  | .69 | Time (T1-T3) | [4.54; 4.63] | 85 | 1.17 | 1 | .283 | .01 | [0.00, 1.00] |
| *Note*. ^1^Reliability is assessed using Cronbach’s Alpha – when alpha is not displayed is because the scale is represented by a single item; ^2^Part Eta Sq = Partial Eta Squared corresponds to the proportion of variance that a variable explains that is not explained by other variables; ^3^CI = Confidence Intervals; in bold significant results. | | | | | | | | | |

**Box Plots**


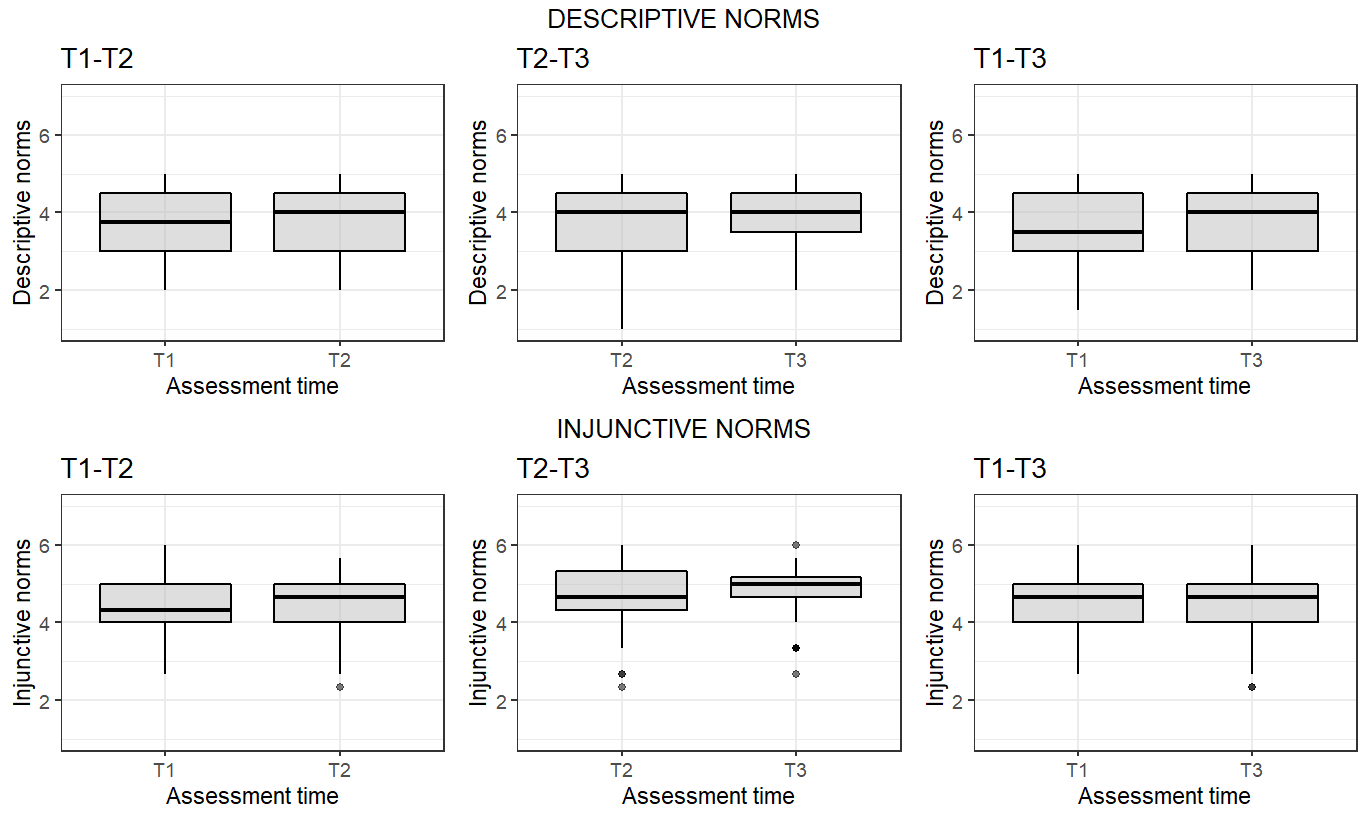


*Note.* In green significant differences. The black horizontal line is the median value.

**Exploratory research question: number of participants reporting flu-like symptoms or positivity to COVID-19 (i.e., positive test) during the two weeks before the assessment.**

| **Time** | **Number of participants reporting flu like symptoms in the previous 2 weeks** | **Number of participants resulting positive to a COVID-19 test in the previous 2 weeks** |
| --- | --- | --- |
| T1 | 19 | 14 |
| T2 | 13 | 0 |
| T3 | 15 | 0 |

1. The hypotheses are reported according to the protocol paper. [↑](#footnote-ref-1)
