# Supplementary material for: Promoting Hand Hygiene During the COVID-19 Pandemic: Parallel Randomized Trial for the Optimization of the Soapp App
Source: JMIR Mhealth Uhealth. 2023 Feb 3;11:e43241. doi: 10.2196/43241 (PMC9938438; doi:10.2196/43241)
Supplement: Multimedia Appendix 3 [file mhealth_v11i1e43241_app3.docx]

**Multimedia Appendix 3**

**Questions from the Interview guide.**

We investigated:

1. how participants describe their views of the app-based intervention as a whole
2. how participants describe their views on barriers and facilitators regarding using the app and how this impacted their hand hygiene in everyday life
3. What suggestions for improvement regarding content or features participants see based on their needs as app-users

(Not all questions were asked in each interview)

Start:

- open ended question “Can you tell me about the last five weeks with the app?”

Intervention fidelity:

- How would you describe the quality of the app? Where do you see areas of improvement?
- To what extent did the app meet your needs? What needs remained unfulfilled?
- Did the app help you to improve your hand hygiene? What exactly did the app facilitate? What barriers/problems remain unsolved?
- To what extent did the app help you in everyday life? What exactly did change? Were you able to implement the tasks in your everyday life?
- How would you describe the usefulness of the given information? Did you feel like the information were believable?
- What’s your opinion of the animated videos? How could we improve them?

Usability:

- Did the app always work as intended (technical aspect)?
- How difficult did you find the app/navigating the app? Is there something we could simplify?
- The aim of this app is in a later stage to be available to the whole population of Switzerland. Do you think that most people are able to operate the app?
- What’s your opinion on the used language level? Was it always understandable or was it sometimes too complicated?
- What’s your opinion on the push notifications? What’s your opinion on the number of push notifications?

Engagement

- How strong was your interest? How did your interest change over the course of the study? Were there contents in which you were not interested?
- How motivated were you to implement the tasks? Was there something that increased your motivation? How could we increase the motivation of other participants?

Adherence

- Were you able to complete every task? Did you skip tasks? Do you think other participants did skip tasks and why/why not?
- In what situation did you skip tasks, what was the reason?
- What’s your assessment, do you think that your answers in the questionnaires were always authentic 🡪 Why/Why not? Do you think that the other participants always answered honestly?
