# Supplementary material for: Promoting Hand Hygiene During the COVID-19 Pandemic: Parallel Randomized Trial for the Optimization of the Soapp App
Source: JMIR Mhealth Uhealth. 2023 Feb 3;11:e43241. doi: 10.2196/43241 (PMC9938438; doi:10.2196/43241)
Supplement: Multimedia Appendix 4 [file mhealth_v11i1e43241_app4.docx]

**Multimedia Appendix 4**

- **Age distribution (mean in red)**


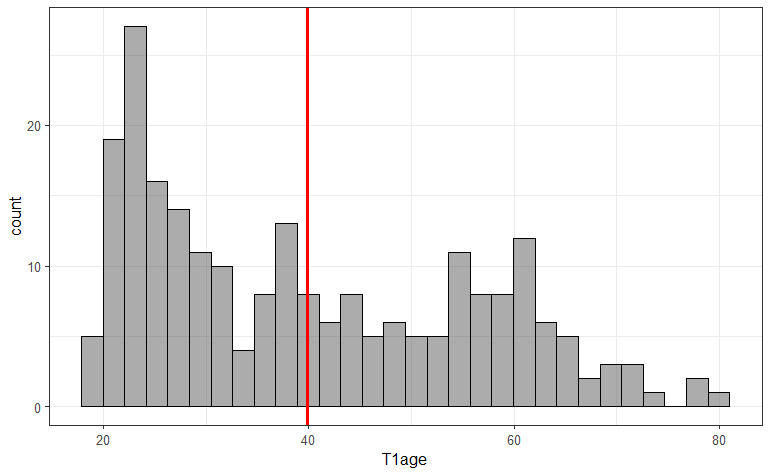


- **Participant characteristics and descriptive statistics on hand hygiene, engagement, usability and satisfaction**

|  | **All** | **H-H** | **H-M** | **H-S** | **M-H** | **M-M** | **M-S** | **S-H** | **S-M** | **S-S** |
| --- | --- | --- | --- | --- | --- | --- | --- | --- | --- | --- |
| **Baseline** |  |  |  |  |  |  |  |  |  |  |
| **Sample size** | 190 | 23 | 21 | 21 | 20 | 22 | 20 | 21 | 19 | 23 |
| **Mean age (SD)** | 39.9  (15.9) | 38.6  (16.8) | 37.0 (14.6) | 42.6 (15.8) | 34.6 (13.2) | 41.1 (16.2) | 44.3 (17.0) | 41.2 (16.1) | 39.5  (17.8) | 39.8 (16.4) |
| **Women (%)** | 139  (73) | 16  (70) | 17  (81) | 18  (86) | 15  (75) | 14  (64) | 14  (70) | 13  (62) | 16  (84) | 16  (70) |
| **High school education (%)** | 129  (68) | 16  (70) | 12  (58) | 14  (67) | 16  (80) | 16  (73) | 14  (70) | 14  (67) | 15  (79) | 12  (52) |
| **Employed (%)** | 101  (53) | 12  (52) | 13  (62) | 11  (52) | 11  (55) | 12  (55) | 11  (55) | 11  (52) | 8  (42) | 12  (52) |
| **Living alone (%)** | 49  (26) | 8  (35) | 4  (19) | 6  (29) | 4  (20) | 7  (32) | 7  (35) | 5  (24) | 3  (16) | 5  (22) |
| **Mean HH (SD)** | 4.01  (.82) | 3.88  (.83) | 4.03 (.69) | 3.95 (.83) | 3.91  (.80) | 4.09  (.73) | 4.12  (.76) | 4.13  (.89) | 4.08  (.98) | 3.92  (.96) |
| **Follow-up** |  |  |  |  |  |  |  |  |  |  |
| **Mean HH (SD) with NA imputation** | 4.18  (.85) | 4.17  (.87) | 4.23  (.71) | 4.10  (.78) | 3.91  (.73) | 4.13  (1.00) | 4.35  (.76) | 4.10  (.01) | 4.21  (.94) | 4.42  (.79) |
| **Mean HH (SD) without NA imputation** | 4.21  (.91) | 4.36  (.69) | 4.52  (.52) | 4.27  (.79) | 3.86  (.72) | 3.99  (1.29) | 4.61  (.42) | 3.77  (1.23) | 4.03  (1.17) | 4.42  (.89) |
| **Mean Engagement (SD)** | 4.42  (.94) | 4.67  (.91) | 4.76  (.73) | 4.82  (.92) | 3.86  (1.04) | 3.95  (.90) | 4.46  (.75) | 4.29  (.68) | 4.53  (1.00) | 4.41  (1.10) |
| **Mean Usability (SD)** | 4.89  (.72) | 5.16  (.66) | 5.07  (.66) | 5.15  (.59) | 4.68  (.71) | 4.70  (.79) | 4.61  (.81) | 4.49  (.85) | 5.14  (.41) | 4.98  (.69) |
| **Mean Satisfaction (SD)** | 2.01  (.55) | 2.13  (.44) | 2.12  (.59) | 2.24  (.42) | 1.75  (.69) | 1.75  (.61) | 2.05  (.43) | 1.80  (.69) | 2.18  (.46) | 2.09  (.43) |
| *Note.* SD = Standard deviation; HH = Hand Hygiene. Intervention groups are specified as it follows: H-H = Habit-Habit; H-M = Habit-Motivation; H-S = Habit-Social; M-H = Motivation-Habit; M-M = Motivation-Motivation; M-S = Motivation-Social; S-H = Social-Habit; S-M = Social-Motivation; S-S = Social-Social. | | | | | | | | | | |

- **Main effects and interactions between modules on hand hygiene behavior at key times, including results from robust ANOVA.**

|  |  |  |  | **Parametric ANOVA** | | | | | **Robust ANOVA** | |
| --- | --- | --- | --- | --- | --- | --- | --- | --- | --- | --- |
| **Hp** | **Outcome** | **Factor** | **N** | **F** | **df** | ***P* value** | **Part Eta Sq^2^** | **95% CI^3^** | **coefficient^4^** | **p-value** |
| H1 - H2 | Hand Hygiene | Group^1^ | 190 | .33 | 8 | .954 | .01 | [0.00, 1.00] | .75 | .646 |
|  |  | Time (T1-T3) |  | **10.95** | **1** | **.001** | **.06** | **[0.01, 1.00]** | **11.71** | **.001** |
|  |  | Time*Group |  | 1.19 | 8 | .306 | .05 | [0.00, 1.00] | .95 | .487 |
| H3 | Hand Hygiene | Habit | 190 | 1.25 | 1 | .265 | .01 | [0.00, 1.00] | 3.03 | .085 |
|  |  | Time (T1-T3) |  | **10.87** | **1** | **.001** | **.05** | **[0.01, 1.00]** | **16.12** | **<.001** |
|  |  | Time*habit |  | 1.07 | 1 | .301 | .01 | [0.00, 1.00] | 1.33 | .251 |
| H4 | Hand Hygiene | Motivation | 190 | .00 | 1 | .995 | .00 | [0.00, 1.00] | .75 | .387 |
|  |  | Time (T1-T3) |  | **10.86** | **1** | **.001** | **.05** | **[0.01, 1.00]** | **14.80** | **<.001** |
|  |  | Time*Motivation |  | .94 | 1 | .332 | .00 | [0.00, 1.00] | .58 | .446 |
| H5 | Hand Hygiene | Social | 190 | .75 | 1 | .387 | .00 | [0.00, 1.00] | 2.21 | .140 |
|  |  | Time (T1-T3) |  | **10.83** | **1** | **.001** | **.05** | **[0.01, 1.00]** | **14.87** | **<.001** |
|  |  | Time*Social |  | .41 | 1 | .522 | .00 | [0.00, 1.00] | .34 | .559 |
|  |  |  |  | **F** | **df** | **p-value** | **Eta Sq^2^** | **95% CI** | **chi-squared^5^** | **p-value** |
| H6 | Engagement | Group (T3) | 148 | **2.19** | **8** | **.03** | **.11** | **[0.01, 1.00]** | 15.40 | .051 |
| H7 | Usability | Group (T3) | 148 | **2.46** | **8** | **.02** | **.12** | **[0.01, 1.00]** | **16.05** | **.04** |
| H8 | Satisfaction | Group (T3) | 148 | 1.46 | 8 | .176 | .11 | [0.00, 1.00] | 12.38 | .135 |
| *Note*. ^1^Group = Intervention group; ^2^Part Eta Sq = Partial Eta Squared corresponds to the proportion of variance that a variable explains that is not explained by other variables; ^3^CI = Confidence Intervals; ^4^robust coefficients from WRS2 R package; ^5^chi-squared value from Kruskasl-Wallis test. | | | | | | | | | | |

- **Box plot for H1 and H2: visual representation of intervention group differences in hand hygiene at baseline and follow-up.**


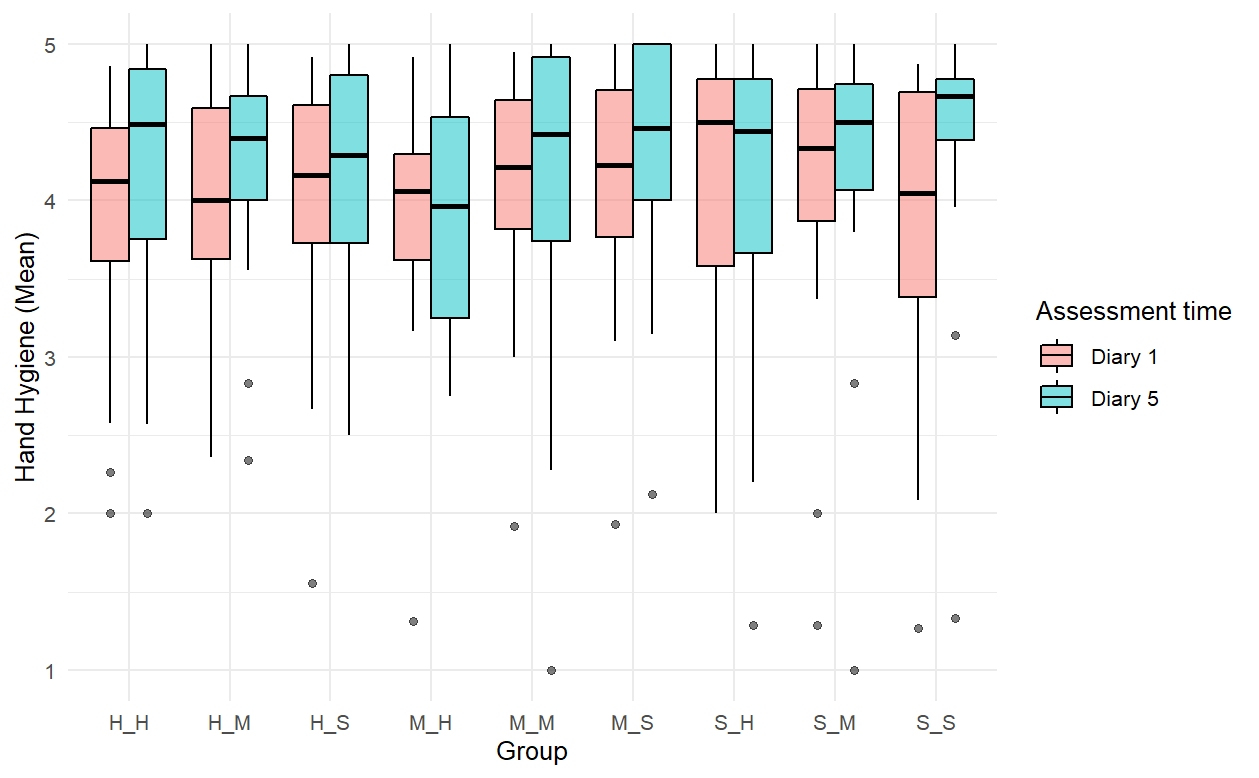


*Note.* The black horizontal line in each box plot represents the median value.

- **Sensitivity analysis for H1-H5.**

| **Main effects and interactions between modules on hand hygiene behavior at key times without LOCF imputation** | | | | | | | | |
| --- | --- | --- | --- | --- | --- | --- | --- | --- |
| **Hp** | **Factor** | **N^1^** | **Parametric ANOVAs** | | | | **Robust ANOVAs** | |
|  |  |  | **F** | **df** | **p-value** | **Part Eta Sq^2^** | **Coefficient** | **p-value** |
| H1  H2 | Int.Group^3^ | 118 | .59 | 8 | .783 | .04 | .58 | .788 |
|  | **Time (T1-T3)** |  | **6.54** | **1** | **<.05** | **.06** | **5.11** | **<.05** |
|  | Time* Int.Group |  | 1.59 | 8 | .135 | .10 | 1.65 | .156 |
| H3 | Habit | 118 | 0.13 | 1 | .723 | .00 | .86 | .357 |
|  | **Time (T1-T3)** |  | **6.29** | **1** | **<.05** | **.05** | **10.61** | **<.01** |
|  | Time*habit |  | 1.14 | 1 | .287 | .01 | 3.65 | .061 |
| H4 | Motivation | 118 | .03 | 1 | .869 | .00 | .27 | .605 |
|  | **Time (T1-T3)** |  | **6.23** | **1** | **<.05** | **.05** | **9.34** | **<.01** |
|  | Time*Motivation |  | .09 | 1 | .093 | .00 | .00 | .975 |
| H5 | Social | 118 | .12 | 1 | .734 | .00 | 1.13 | .292 |
|  | **Time (T1-T3)** |  | **6.23** | **1** | **<.05** | **.05** | **9.22** | **<.01** |
|  | Time*Social |  | .05 | 1 | .823 | .00 | .07 | .788 |

*Note*. ^1^ N corresponds to participants who filled out the hand hygiene diary at T1; ^2^ Part Eta Sq = Partial Eta Squared corresponds to the proportion of variance that a variable explains that is not explained by other variables; ^3^ Int.Group = Intervention group.
