# Supplementary material for: Promoting Hand Hygiene During the COVID-19 Pandemic: Parallel Randomized Trial for the Optimization of the Soapp App
Source: JMIR Mhealth Uhealth. 2023 Feb 3;11:e43241. doi: 10.2196/43241 (PMC9938438; doi:10.2196/43241)
Supplement: Multimedia Appendix 5 [file mhealth_v11i1e43241_app5.docx]

**Multimedia Appendix 5**

| **Table 1. Summary of the themes and subthemes that developed from the thematic analysis.** | |
| --- | --- |
| **Themes and subthemes^1^** | **Description** |
| User experience and app functionality | Features that appeal to the user (i.e., visual design features) and enable a simple and intuitive use of the app without technical barriers (i.e., technical smoothness). |
| Importance of guidance | A clear guidance that enables users to understand and act in accordance with the app and the proposed tasks. To this aim, the app should provide the right amount of guidance without overwhelming users with too many notifications. |
| Variety and timeliness of the task load | Variety in the daily engagement with the tasks (e.g., different workloads on different days) and the right timing for the allocation of content (e.g., more tasks towards the end of the intervention to stay engaged). |
| Reasons for participation | Initial reasons for taking part in the study (e.g., learning something new) and users' motivation to continue participating (e.g., feeling obligated to finish the study). |
| Change in awareness of hand hygiene and its implications | Features that create awareness of hand hygiene (e.g., diary days, key situations) and thereby lead to a change in behavior and in the perception of the issue and its implications. |
| Social interaction | Features that leverage social interaction in different ways. |
| *Personal communication and connectedness* | Certain human elements of the app give the user a sense of authenticity, so they do not feel like they are interacting with a machine. Additionally, features that allow the user to feel connected to others leads to a sense of community. |
| *Social comparison* | Features that allow users to compare themselves to others in terms of implementation of hand hygiene. |
| Wish for personalization | Content lacking personal relevance leads to the user wishing for features which allow them to tailor the app more closely to themselves. |
| *Note.* ^1^ = Subthemes are in italics | |

| **Table 2. Additional quotes for each of the emerged themes** |
| --- |
| **User Experience and App Functionality** |
| *Because it is a simple app. It is easy to use for everyone and everything is always nicely described.* [P4, Habit-Motivation, High Adherence] |
| *In the beginning, I participated pretty motivated, but then my motivation actually dropped quite a bit after these technical problems I had when I could not manage to download these images.* [P8, Social-Habit, Low Adherence] |
| *If you could make it a little more over-seeable and a little less long, maybe then more people would use the app.* [P9, Motivation-Motivation, Low Adherence] |
| *But maybe you could have colored the headings a little bit, or put a picture in between – I mean, why not.* [P6, Social-Social, Middle Adherence [general comment about the app / intervention]] |
| *The process of downloading and logging into the app - that I found very easy, without any complications.* [P1, Habit-Habit, Middle Adherence] |
| *I found the handling [of the app] good, practical…* [P7, Habit, Habit, Middle Adherence] |
| *It is a simple app in which you could enter your answers - there was certainly no problem dealing with the app…* [P9, Motivation-Motivation, Low Adherence] |
| *I had the feeling that there was still a bit of a technical hitch.* [P3, Social, Habit, Low Adherence] |
| *At the beginning it took some time to get used to - sometimes, it said today's task, and then I wanted to click on it, but somehow that did not work. Until I realized that the app needs some time until it accepts it.* [P7, Habit, Habit, Middle Adherence] |
| *The design of the app is - is still in an early stage. And maybe it would not have been bad to use - how do you say - to emphasize some things in the design, to mark something in bold or in color, something like that.* [P8, Social-Habit, Low Adherence] |
| *In the beginning, I participated pretty motivated, but then my motivation actually dropped quite a bit after these technical problems I had when I could not manage to download these images.* [P8, Social-Habit, Low Adherence] |
| **Importance of Guidance** |
| *It [the app] has certainly made you feel secure. Just in the sense that at certain - at certain points, where you perhaps did not even think of it yourself, when you had to do something, it provided you with input or ideas like “perhaps this is something you could do as well” - I have not even thought of that - or “there are still times when you should stick to it more often” and so. That was certainly a great benefit for me.* [P7, Habit-Habit, Moderate Adherence] |
| *…and I knew I had to do something, but what?* [P2, Habit-Habit, High Adherence] |
| *Sometimes it was not quite clear to me where I could find - what I have to do if the app does not show me anything-* [P3, Social-Habit, Low Adherence] |
| *For me, they [the instructions] are clearly understandable, simple, not too much or too long. If there would have been a two-page text, then I would have put it down. I never had the feeling that something was too long, I never felt like I not wanting to read it or like losing interest in it.* [P7, Habit-Habit, Middle Adherence] |
| *Because for me, it was just - yes, I understood what I had to do. I feel like it was all good.* [P6, Social-Social, Middle Adherence] |
| *And yes, as I said, in the end I was puzzled when it said that it was already finished. That happened somehow relatively quickly and that it also said "you can uninstall the app now", because I thought okay, then maybe you could have just kept running it.* [P8, Social, Habit, Low Adherence] |
| *Afterwards in the evening you had several such messages on your phone - and then I did not know what to do anymore - do I have to catch up with them?* [P3, Social, Habit, Low Adherence] |
| **Variety and Timeliness of the Task Load** |
| *It is funny, my motivation dropped relatively quickly. It was on a high level - because I like to try out new things, I had the feeling that I was very motivated when I started. But then I felt like it was probably always going to be the same now, and there is never anything new. So, then my motivation sank.* [P3, Social-Habit, Low Adherence] |
| *Otherwise, you start to think "when will there be another [question]?" and then you suddenly have no interest in doing it anymore. If you could do it more regularly, more diligently, then it much easier, at least that is the case with me.* [P6, Social-Social, Middle Adherence] |
| *Well, I still would have found it somehow exciting if it had contained a few more exercises. I can’t tell you on the spot what kind of exercise, but yes, I would have liked that.* [P7, Habit-Habit, Middle Adherence] |
| *What I described as positive before, the fact that it's always the same, also has its downside. This makes it very much a routine, and then you- at least that's what happened to me- that it eventually wears off and you don't really notice it anymore, so to speak.* [P3, Social-Habit, Low Adherence] |
| *I didn't have the feeling that there were so many [push notifications]. Especially towards the end I became unsure of whether everything is still running correctly. It wouldn’t have bothered me if there had been more, maybe another reminder or something. Overall, it was certainly not too much, for me it was a good range- or it could have been even two, three times more, then it would have been okay.* [P7, Habit-Habit, Middle Adherence] |
| **Reasons for Participation** |
| *I am generally very interested in trying out new things and see what is new on the market, or what different things are currently being tested. And so, I was very curious.* [P3, Social-Habit, Low Adherence] |
| *So, in general I didn’t have any expectations, I just let myself be surprised. I have an app- this Instagram app- and then this information always came up, and then I thought, that makes me curious, so I clicked on it.* [P2, Habit-Habit, High Adherence] |
| *At the beginning my interest was strong because I thought “yes something new, I have never heard something like this before”.* [P4, Habit-Motivation, High Adherence] |
| *Well, I am retired, I am interested in studies, I studied psychology myself a long time ago, but then I did not work in this profession, and I like to take part in surveys.* P9, Motivation-Motivation, Low Adherence] |
| *So, on the one hand certainly something to go along for the protection and health* [P7, Habit-Habit, Middle Adherence] |
| **Change in Awareness of Hand Hygiene and its Implications** |
| *There were moments in between when I thought, "hey, I can still wash my hands without writing it down," but afterwards it hit me – I realized “now you have not done it three times”* [P6, Social-Social, Moderate Adherence] |
| *What has remained is that I wash my hands much more than before the pandemic.* [P9, Motivation-Motivation, Low Adherence [general comment about the app / intervention]] |
| *It is almost scary when you watch this video, how many places that actually, that you - it has bacteria everywhere. I did not know that - that it really is everywhere. I actually only became aware of that when I watched this video. So, the video, I have to say, it is good.* [P4, Habit, Motivation, High Adherence [commenting on the video about beliefs and consequences from the motivation module]] |
| *Perhaps also being more concerned about health than before.* [P6, Social, Social, Middle Adherence [general comment about the app / intervention]] |
| *I used to go out a lot, or play with the kids, the kids from the neighborhood, but it never occurred to me to wash my hands after that. That teaches you - that was good of course.* [P6, Social-Social, Middle Adherence [commenting on self-report questions and key times]] |
| **Social Interaction – Subtheme 1: Personal Communication and Connectedness** |
| *I really felt addressed by the tasks. The tone wasn’t lecturing at all. Not like “now you must do this, and then you have to do that”.* [P2, Habit-Habit, High Adherence] |
| *And I felt like- I was really touched by this care and regardfulness in handling things [inside the app]. This dialogical way of dealing with each other. This was very, very important to me personally. And I didn't regret for a second that I had taken part, really.* [P1, Habit-Habit, Middle Adherence] |
| *[I would have appreciated] a short video with other participants who then motivate you, because statistics and reading news is something else than someone speaking directly to you.* [P8, Social-Habit, Low Adherence] |
| *Would have preferred more interaction with other participants? [Interviewer]. No, I believe- no, I wouldn’t find this reasonable. Somehow a threshold would be crossed - so you can't communicate everything with our cell phone- you need to be able to live somehow. No, that would be too much.* [P5, Motivation-Social, High Adherence] |
| **Social Interaction – Subtheme 2: Social Comparison** |
| *...I showed my results because I thought at the beginning that is funny, but then it makes - well, you have no relation to these people, there is no real interaction. So, you only see that others are also at it, but that was actually already clear to me without having to set a so-called "range" in the evening. I now have this 95 or 97 percent, but that did not convince me. It did not increase my motivation, it had rather contributed to the fact that a fatigue occurred. [P5, Motivation-Social, High Adherence [commenting on scoreboard from the social module]]* |
| *...and then the result comes out, then I also take note of that to see how I stand there compared to others. [P9, Motivation, Motivation, Low Adherence [general comment about the app / intervention]]* |
| *What did you like most about the app?* [Interviewer] *That there were more who participated, and you could see how they were doing.* [P6, Social-Social, Moderate Adherence] |
| **Wish for Personalization** |
| *So, for example, that when I am asked regularly over the course of four weeks if I washed my hands before I take my contact lenses out - if I am no lens wearer, then that question starts to annoy me over time.* [P5, Motivation-Social, High Adherence] |
| *What actually annoyed me about the whole thing is - it always said, "hand hygiene at key times", and these key times, I found, they just did not fit my lifestyle at that time. [P9, Motivation-Motivation, Low Adherence [commenting on self-report questions and key times]]* |
| *…or I then there were certain questions that - that to me were not relevant at all. [P7, Habit-Habit, Middle Adherence [commenting on self-report questions and key times]]* |
| *And certainly, more motivating, I can imagine. [Interviewer]*  *Yes, yes exactly. If you can tailor it to yourself, and also if certain things can be omitted that you do not apply to you at all. [P5, Motivation, Social, High Adherence [commenting on self-report questions and key times]]1* |
| *Just the importance of the key moments and this personalization, that is what I find most important, that would give the app an even stronger expression.  [P5, Motivation, Social, High Adherence [commenting on self-report questions and key times]]* |
| *At the beginning you could say you do not have children and therefore you will not go to the toilet with children or things like that. That afterwards I only get asked about these situations or inputs that are really relevant for me. So that you can maybe adapt it even more personally to your own habits or circumstances.* [P3, Social, Habit, Low Adherence [commenting on self-report questions and key times]] |
| *There were these action plans that you could create when you - […] or what would be practical is, if in between - that happened very rarely that there was a reminder like "now would be time for" - so there are times when you should also remember other things. Maybe it would have been handy to have a reminder like "did you think of that?" for other situations as well. [P7, Habit, Habit, Middle Adherence [commenting on action planning and reminder from the habit module]]* |
